# Three-Enzyme Cascade Catalyzes Conversion of Auramycinone to Resomycin in Chartreusin Biosynthesis

**DOI:** 10.1021/acschembio.5c00205

**Published:** 2025-07-02

**Authors:** Magdalena Niemczura, Aleksi Nuutila, Rongbin Wang, Katariina Rauhanen, S. Eric Nybo, Mikko Metsä-Ketelä

**Affiliations:** † Department of Life Technologies, University of Turku, FIN-20014 Turku, Finland; ‡ Department of Pharmaceutical Sciences, College of Pharmacy, 1701 Ferris State University, Big Rapids, Michigan 49307, United States

## Abstract

Chartreusin is a potent antiproliferative agent that contains a unique aromatic pentacyclic bislactone carbon scaffold. The biosynthesis of type II polyketide aglycone has been extensively investigated and shown to proceed through a tetracyclic anthracycline intermediate. The last remaining unknown steps are the conversion of auramycinone to resomycin C. Here we have discovered three enzymes that play crucial roles in two mechanistically distinct dehydration reactions. We show that ChaX is an NAD­(P)­H-dependent auramycinone quinone reductase that allows the cyclase-like ChaU to catalyze the formation of 9,10-dehydroauramycinone via a carbanion intermediate. In contrast, the cyclase-like ChaJ, homologous to ChaU, is responsible for subsequent 7,8-dehydration via a canonical carbocation intermediate, yielding resomycin C. The results were confirmed via assembly of the biosynthetic pathway for production of resomycin C in *Streptomyces coelicolor* M1152Δ*matAB*. The work expands the catalytic repertoire of the SnoaL protein family, which has previously been associated with anthracycline fourth-ring cyclization and two-component 1-hydroxylation.

## Graphical abstract

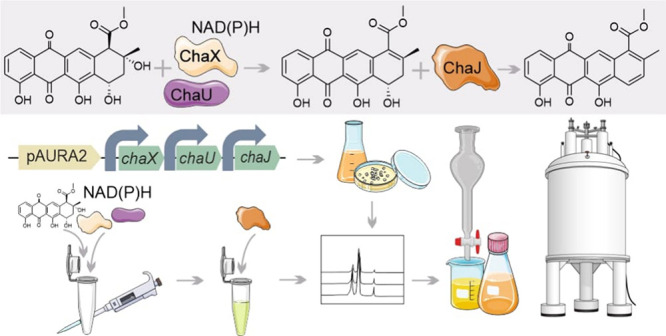

Chartreusin (**1**, Figure 1A) is an aromatic polyketide glycoside produced by *Streptomyces chartreusis*, which was discovered as an antibiotic effective against selected Gram-positive bacteria.^1^ Compound **1** consists of an atypical polyketide-derived bislactone chartarin (**2**) aglycone unit, which is coupled to fucose and digitalose carbohydrate moieties.^2^ Further investigations into the bioactivity revealed **1** to harbor significant anticancer activity, which was revealed to be mediated through single-strand DNA breaks.^3^ The natural congener of **1**, the aminoglycosylated elsamicin A (**3**, Figure 1A) and the semisynthetic derivative, IST-622,^4,5^ have reached phase II clinical trials for the treatment of breast cancer.^6^

**Figure 1:**
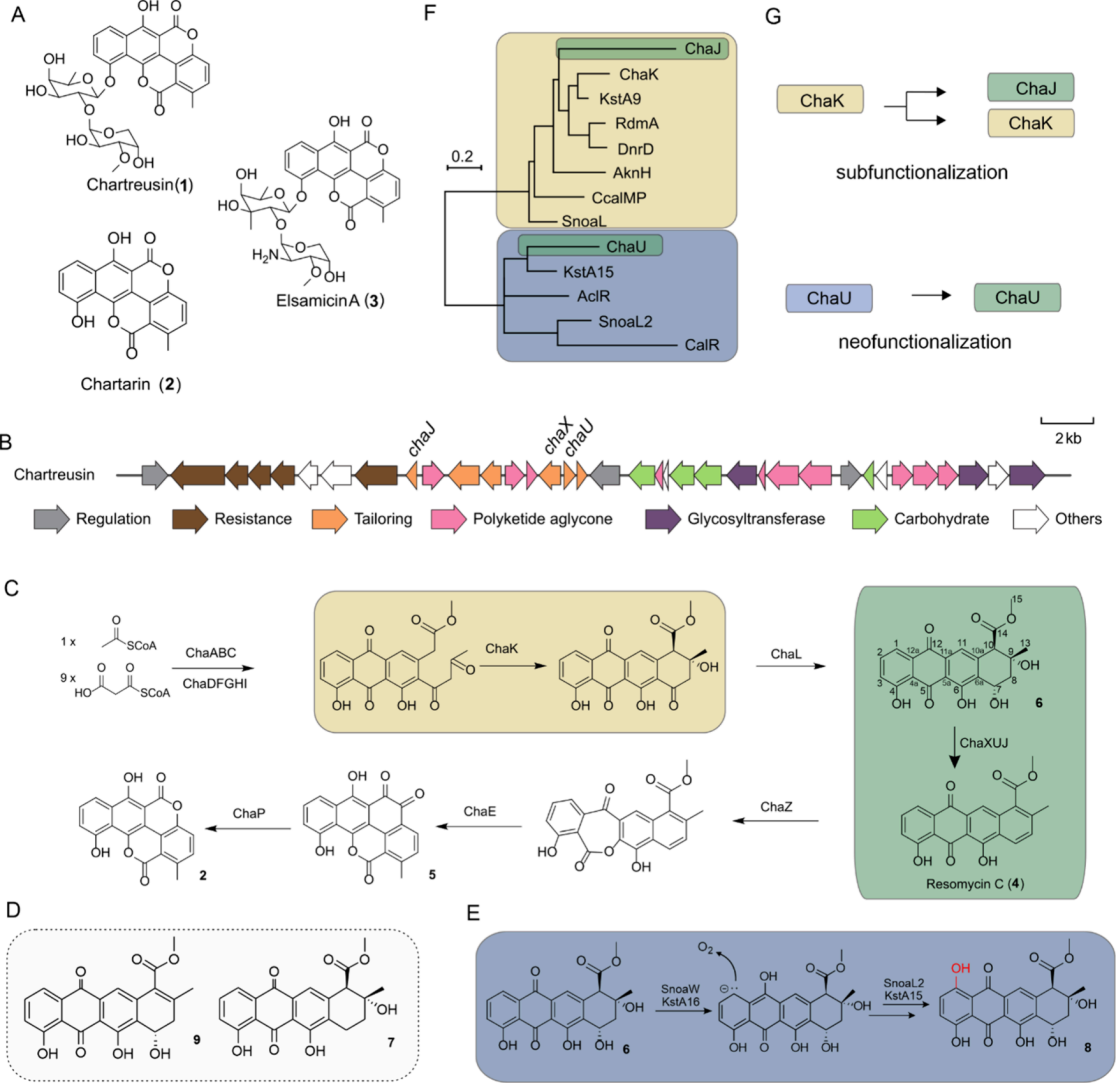
Overview of chartreusin biosynthesis and analysis of the biosynthetic gene cluster. (A) Chemical structures of chartreusin (**1**), chartarin (**2**), and elsamicin A (**3**). (B) Organization of the chartreusin BGC. (C) Chartarin biosynthesis proceeds via initial formation of an anthracyclinone intermediate by the putative fourth ring cyclase ChaK (yellow). The steps leading from auramycinone (**6**) to resomycin (**4**) have been unknown (green). The bislactone chartarin core (**2**) is formed in further tailoring steps from **4**. (D) Structures of 7-deoxyauramycinone (**7**) and 9,10-dehydroauramycinone (**9**). (E) The nogalamycin and kosinostatin pathways encode two-component mono-oxygenase systems SnoaL2/SnoaW and KstA15/KstA16, respectively, for anthracycline 1-hydroxylation. (F) Phylogenetic tree of cyclase-like proteins ChaK (yellow), ChaU (green), and ChaJ (green) from the chartreusin biosynthetic pathway together with known fourth ring cyclases (yellow) and 1-hydroxylases (blue). Legend: SnoaL and SnoaL2 originate from the nogalamycin pathway; KstA9 and KstA15 from the kosinostatin pathway; AknH and AclR from the aclacinomycin pathway; CcalMP and CalR from an uncharacterized anthracycline pathway; RdmA from the rhodomycin pathway; DnrD from the daunorubicin pathway. (G) subfunctionalization of ChaK into ChaK and ChaJ and neofunctionalization of ChaU.

The unique chemical structure has attracted detailed investigations into the biosynthesis of **1**. Isolation of the chartreusin biosynthetic gene cluster (BGC) revealed a canonical type II polyketide synthase pathway with high similarity to anthracycline BGCs (Figure 1B).^7^ The shared evolutionary history was evident from conservation of genes for early steps of the biosynthesis, including the ketosynthase α and β heterodimer (KSα/KSβ) and acyl carrier protein (ACP) for synthesis of the decaketide backbone (Figure 1C). In addition, genes for folding of the reactive poly-β-ketone by various cyclases (CYC), aromatases (ARO), oxygenases (OXY), and methyltransferases (MET) were conserved.^8–10^ Particularly noteworthy was the identification of ChaK (Figure 1C), homologous to unique anthracycline fourth ring cyclases, such as SnoaL,^11^ AknH,^12^ and DnrD^13^ from the nogalamycin, aclacinomycin, and daunorubicin pathways, respectively, that complete the biosynthesis of linear anthracyclinone aglycones. The anthracycline origin of **1** was confirmed through gene inactivation experiments that led to isolation of a tetracyclic resomycin C (**4**) pathway intermediate (Figure 1C).^7,14^

Recent studies have shed light on the conversion of linear tetracyclic intermediate **4** to pentacyclic aglycone **2** (Figure 1C). The flavoenzyme ChaZ has been confirmed as a Baeyer–Villiger monooxygenase that utilizes **4** as a substrate and in conjunction with an NADPH-dependent ketoreductase, ChaE, is sufficient to promote formation of the pentacyclic intermediate **5**.^15^ The vicinal oxygen chelate superfamily enzyme ChaP completes the biosynthesis of **2** in the presence of flavin-activated oxygen.^16^

The remaining unanswered questions regarding the biosynthesis of **2** are the steps leading to the formation of **4**. The anthracycline auramycinone (**6**) has been presumed as the chartreusin pathway intermediate, since both **4** and 7-deoxyauramycinone (**7**) (Figure 1D) have been isolated from *Streptomyces* sp. GW71/2497.^14^ Discourse on whether the two dehydration reactions required for conversion of **6** to **4** occur during isolation of the natural products or are enzyme catalyzed has been inconclusive.^17,14^

The majority of gene products in the chartreusin BGC have been assigned functions,^7^ but curiously the pathway encodes proteins of unknown function with high sequence similarity to two-component anthracycline 1-hydroxylase systems (Figure 1E).^18,19^ Recent mechanistic studies from the nogalamycin and kosinostatin pathways have revealed that atypical SDR (short-chain dehydrogenase/reductase) enzymes (SnoaW/KstA16) catalyze NAD­(P)­H-dependent quinone reduction, which allows the reduced anthracycline to react with molecular oxygen in a manner similar to flavin.^19,20^ The reaction cascade is resolved to 1-hydroxylated products through the action of fourth ring cyclase-like proteins (SnoaL2/KstA15).^19^ The existence of a SDR enzyme, ChaX, and two additional cyclase-like proteins, ChaU and ChaJ, on the chartreusin pathway (Figure 1B) is unexpected since **1** is not hydroxylated in the equivalent position.

Here we demonstrate that ChaX and ChaU catalyze 9,10-dehydration, which represents a third function for enzymes of the SnoaL family of proteins. We further show that the second 7,8-dehydration is catalyzed by cofactor independent ChaJ, without the need for the SDR enzyme ChaX. We further confirmed the enzymatic studies by metabolic engineering and assembly of the chartreusin pathway in *Streptomyces coelicolor* M1152Δ*matAB* for production of **4**.

We initiated the study by phylogenetic analysis of the cyclase-like proteins from the chartreusin BGC together with known fourth ring cyclases and 1-hydroxylases (Figure 1F). Representative proteins were aligned using MUSCLE (Figure S1)^21,22^ after which a maximum likelihood phylogenetic tree was constructed using PhyML+LG^23^ model in SeaView 5.^24^ The cyclase-like proteins clustered in two groups, one of which contained fourth ring cyclases including SnoaL, AknH, and DnrD. Two proteins from the chartreusin BGC, the putative fourth ring cyclase ChaK and a protein of unknown function, ChaJ, were included in this clade. In turn, the cyclase-like enzymes involved in C1-hydroxylation, such as SnoaL2 and KstA15, clustered in the other group, which also contained ChaU from the chartreusin pathway.

To experimentally investigate the function of the proteins, ChaX, ChaU, and ChaJ were produced recombinantly in *Escherichia coli* as N-terminally hexahistidine tagged proteins (Figure S2). However, ChaX could not be produced in soluble form; therefore, initial tests were carried out with cell-free *E. coli* lysates. We surmised that ChaX and KstA16 might have orthologous functions in reduction of the quinone unit of anthracyclinones. This was confirmed in activity assays where both KstA16 and the cell-free extract of ChaX converted **6** into 1-hydroxy-auramycinone (**8**) (Figure 1E) in the presence of the cyclase-like KstA15, NADH, and an NAD­(P)H regeneration system consisting of glucose and glucose dehydrogenase (Figure 2A, traces V and VI). Interestingly, when the ChaX lysate or KstA16 was incubated with ChaU and NADH, we observed the formation of a new product **9** when **6** was used as a substrate (Figure 2A, traces VIII and IX). We utilized the combination of KstA16 and ChaU to convert **6** into **9** in preparative scale for structure elucidation by HR-MS and NMR (Figures S3–S9), which confirmed that the molecule is 9,10-dehydroauramycinone (**9**). The position of the double bond in **9** was determined based on 2D-NMR measurements. COSY and phase sensitive HSQC spectra (Figure S8) and the coupling pattern in the ^1^H-spectrum indicated a CH_2_ carbon at position C8 adjacent to a secondary alcohol at position C7. In comparison to **6** (Figure S10), two additional carbon–carbon double bonded signals, which show HMBC correlations to H11 and H13, are present in the ^13^C-spectrum of **9**. The reaction was fully dependent on the presence of NAD­(P)H (Figure S11). The cell-free lysate of *E. coli* TOP10 without heterologous protein expression was used in negative control reactions to exclude the possibility that native proteins from *E. coli* participated in the reactions (Figure 2A, traces IV, VII, and X). The experiments indicated that ChaX and KstA16 have identical functions, and consequently, KstA16 was used in place of ChaX in all subsequent enzyme assays.

**Figure 2:**
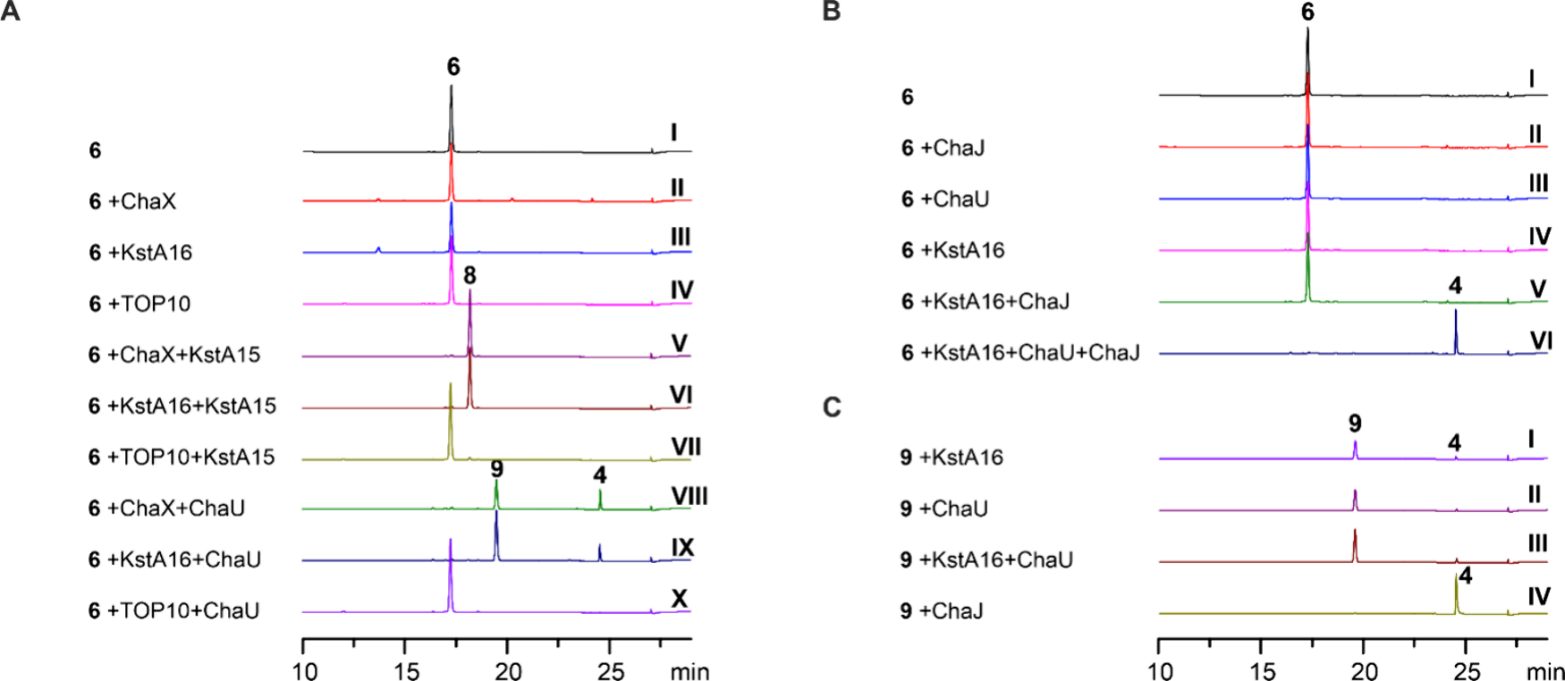
UPLC analysis of enzymatic assays. All chromatograms are recorded at 254 nm. (A) Comparison of the functions of ChaX and KstA16. Reactions were supplemented with NADH and NAD­(P)H regeneration systems. Reactions labeled with ChaX and TOP10 are supplemented with crude lysate of ChaX-TOP10 (ChaX) and crude TOP10 lysate (TOP10). (B) Reactions with KstA16, ChaU, ChaJ, and NADH with auramycinone (**6**) as substrate. (C) Reactions with KstA16, ChaU, ChaJ, and NADH with 9,10-dehydroauramycinone (**9**) as substrate.

Next we examined the functions of ChaU and ChaJ together with KstA16 and NADH in different combinations with **6** as the substrate (Figure 2B). ChaU, ChaJ, and KstA16 alone or the combination of KstA16 and ChaJ did not show activity on **6** (Figure 2B, traces II–V). The combination of KstA16, ChaU, and ChaJ together with NADH converted **6** into a new product (Figure 2B, trace VI), which was verified as resomycin C (**4**) based on HR-MS and NMR methods (Figure S3 and Figures S13–S18). The structure of resomycin C was confirmed based on comparison to chemical shifts reported in the literature^25^ and the appearance of signals for H7 and H8 in the aromatic range of the NMR spectrum at 8.50 and 7.56 ppm, respectively (Tables S3 and S4). The structure assignment for **4** was further supported by the loss of the geminal coupling constant (^2^ *J*_H8a,H8b_ = −15.0 Hz) present in **6** at C8.

To further investigate the reaction cascade, we isolated the intermediate **9** and assayed the compound with ChaU, ChaJ, and KstA16 and NADH (Figure 2C). Incubation with KstA16 and ChaU did not lead to the formation of new products either together or individually (Figure 2C, traces I–III). However, ChaJ converted **9** into **4** in the absence of any cofactors (Figure 2C, trace IV). During our enzymatic assays with **9**, we additionally observed that the second dehydration reaction to **4** also occurs nonenzymatically under acidic conditions. We confirmed the finding by overnight incubation of **9** in 1% HCl, which lead to full conversion to **4** (Figure S12). We surmise that the detection of minor quantities of **4** in some of the enzyme reactions (e.g., Figure 1A, traces V–VI) is due to the instability of the intermediate **9**. Together these results indicate that the NAD­(P)H dependent reductase ChaX and cyclase-like ChaU catalyze a 9,10-dehydration of **6**, after which the cofactor independent cyclase-like ChaJ catalyzes a second 7,8-dehydration, resulting in the formation of **4**.

To confirm the biological relevance of our finding, we assembled the biosynthetic pathway for the formation of **4** in *S. coelicolor* M1152Δ*matAB* using synthetic BioBricks parts (Table S1). To improve the yields of **6**, we first modified our previously cloned construct pEN10003 (Figure 3A), which contained early biosynthetic genes from the nogalamycin and aclacinomycin pathways, but which only produced 0.94 mg/L of **6** in SG-TES media.^26^ Plasmid pAURA2 was constructed with ribozyme insulators and terminator sequences to enhance mRNA stability (Figure 3A).^27^ Using our previous strategy,^28^ the strong SP41 promoter was fused to the *ltsvJ* insulator, driving the expression of the minimal polyketide synthase *snoa123.* Promoter-insulator SP42*-vtmoJ* controlled expression of *snoaDEMB* for biosynthesis of a tricyclic intermediate, and promoter insulator SP44*-riboJ* specified transcription of *aknHGU* to produce **6** (Figure 3A). The construct was cloned into pOSV821, which incorporates a 5′-fd terminator and 3′-ttsbiA terminator^28^ (Table S2). The expression of pAURA2 resulted in an increase in the production of **6** at 77.78 mg/L in SG-TES media when it was expressed in *Streptomyces coelicolor* M1152Δ*matAB*. We confirmed the production of **6** via HRMS (Figure S19)

**Figure 3:**
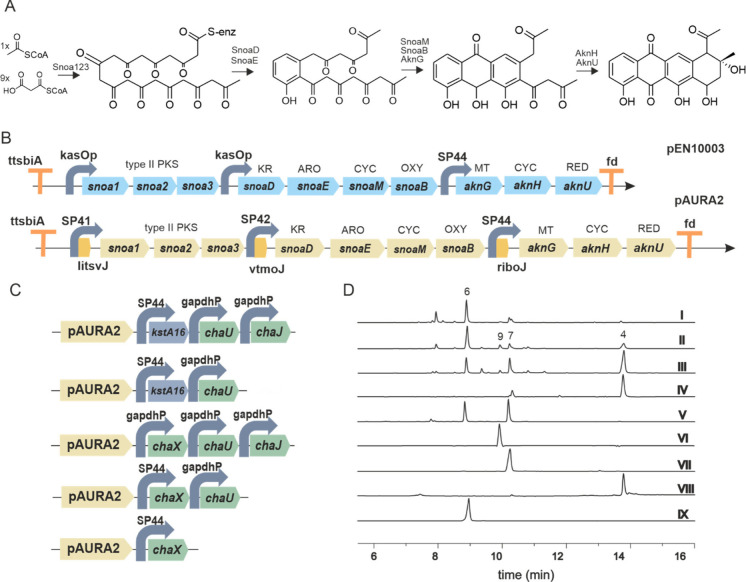
Assembly of the resomycin C biosynthetic pathway in *Streptomyces coelicolor* M1152Δ*matAB*. (A) Production of auramycinone (**6**) was increased via the use of genetic insulators LitsvJ, VtmoJ, and RiboJ. The previously described low yield producing plasmid pEN10003 was engineered to the high yield producing pAURA2. Legend: KR = ketoreductase, ARO = aromatase, CYC = cyclase, MT = methyltransferase, and RED = reductase (B) SBOL diagrams for the BioBrick expression constructs used to study the functions of *kstA16*, *chaX*, *chaU*, and *chaJ*. (C) UPLC chromatogram traces recorded at 450 nm of culture extracts. Legend: I, *Streptomyces coelicolor* M1152ΔmatAB_pAURA2; II, pAURA2_kstA16 chaU; III, pAURA2_k16 chaU chaJ; IV, pAURA2 chaU chaX chaJ; V, pAURA2_chaX; VI, purified 9,10-dehydroauramycinone (**9**); VII, purified 7-deoxyauramycinone (**7**); VIII, purified resomycin C (**4**); IX, purified auramycinone (**6**).

We assembled the *chaU*, *chaX*/*kstA16*, and *chaJ* genes into a second integrating vector, pENTG3, and expressed them in different combinations as BioBricks gene cassettes (Figure 3B). The genes were expressed under the natural *gapdhP* or the strong synthetic SP44 promoter. Expressing *kstA16* together with *chaU*, yielded small amounts of **7**, **4**, and **9** (Figure 3C). Upon addition of *chaJ*, we observed an increase in the yield of **4**; however, the conversion was not complete (Figure 3C). Switching *kstA16* to the native *chaX* gene and expressing it together with *chaJ* and *chaU* resulted in almost complete conversion of **6** to **4**. Expression of *chaX* alone led to the formation of 7-deoxyauromycinone (**7**) (Figure 1D), which was identified by HR-MS and NMR measurements (Figure S3 and Figures S20–S26). The presence of signals for two methylene groups at both C8 and C7 in **7** confirmed the absence of any other substituents at these positions, which proved the loss of the hydroxyl group at C7. The orthologous enzymes SnoaW^19^ and KstA16 ^20^ have been noted to harbor equivalent activity *in vitro* under anoxic conditions in the presence of the cyclase-like partner proteins. Assaying **7** with KstA16, ChaU, ChaJ, and NADH indicated that it is a nonreactive shunt product (Figure S27).

The experiments described above let us propose a possible mechanism for the two dehydration reactions. In the first dehydration step, the SDR enzyme ChaX uses NAD­(P)H to donate a hydride to the C5 carbonyl of the substrate **6** (Figure 4A) similarly to SnoaW and KstA16.^19,20^ In nogalamycin and kosinostatin biosynthesis, the negative charge delocalizes over the anthraquinone ring system and the carbanion at C1 leads to a reaction with molecular oxygen.^19,20^ In chartreusin biosynthesis, we propose that molecular oxygen attacks the chemically equivalent C11 position. The resulting peroxy anion then abstracts the adjacent H11 proton, leading to the removal of the C9 hydroxyl group and formation of the double bond between C9 and C10 through an E1cB type elimination reaction (Figure 4A). To finalize the formation of **9**, the C11 peroxyl group deprotonates the adjacent C12 phenol and leaves as hydrogen peroxide leading to the formation of **9**. Supporting this hypothesis, the addition of ChaU did not quench peroxide formation in a reaction of **6** with KstA16 (Figure S28), which is in contrast to 1-hydroxylation where the inclusion of SnoaL2 prevents accumulation of H_2_O_2_.^18^

**Figure 4:**
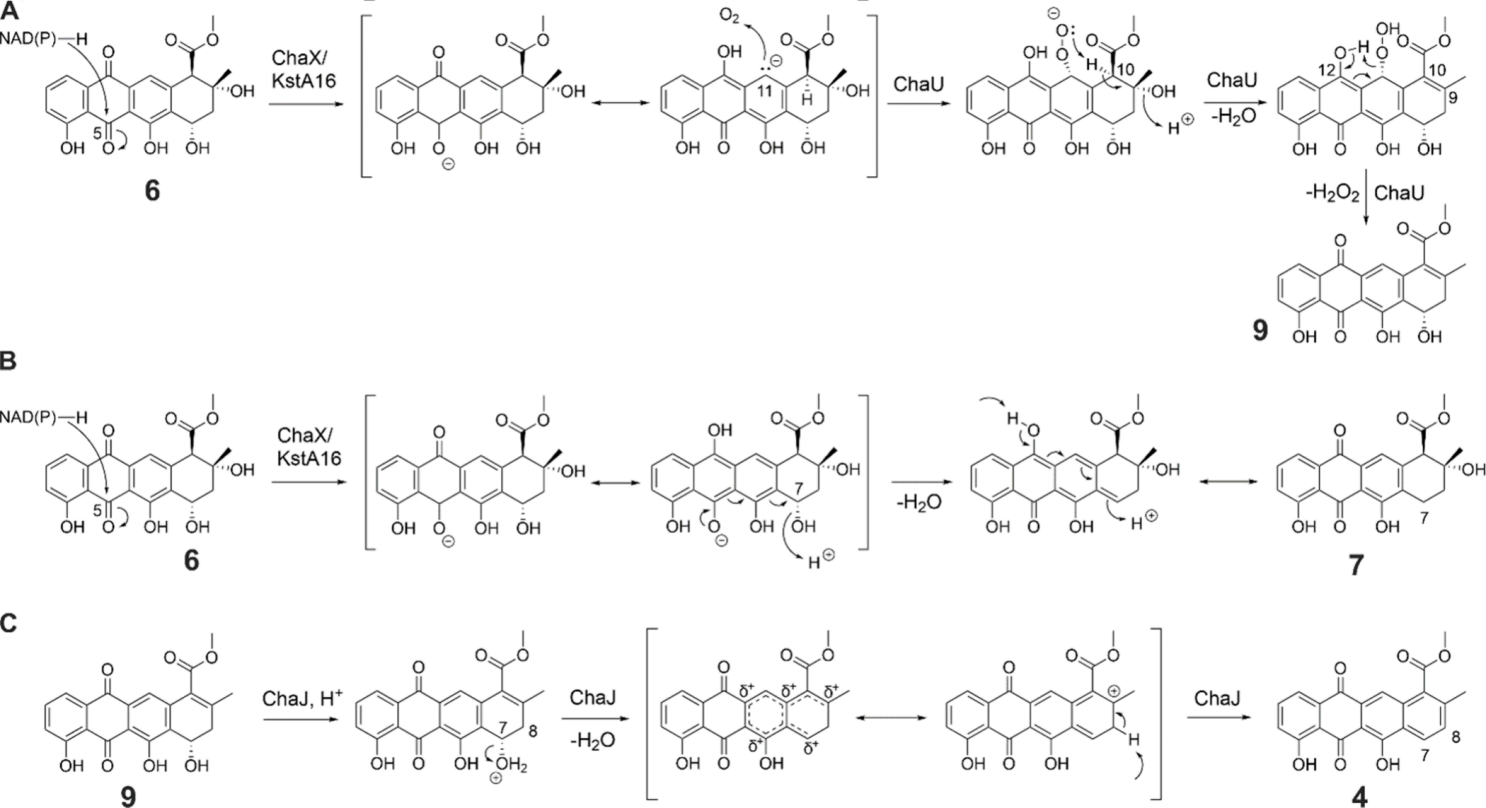
Proposed reaction mechanisms of ChaX, ChaU, and ChaJ. Putative reaction mechanism for (A) 9,10 dehydration catalyzed by ChaX and ChaU, (B) formation of the 7-deoxygenation shunt product, and (C) 7,8 dehydration catalyzed by ChaJ.

In the absence of ChaU, the *in vivo* experiments led to formation of **7** by ChaX. We propose that the formation of this shunt product proceeds through the same mechanism as has been proposed for SnoaW,^19^ which involves the generation of a quinone methide intermediate that may rearrange to **7** (Figure 4B).

In the second dehydration reaction, we propose that ChaJ catalyzed 7,8-dehydration of **9** into **4** proceeds through an E1 mechanism (Figure 4C). The protonation of the hydroxyl group at C7 generates a good leaving group and the resulting secondary carbocation is delocalized over the aromatic B-ring and over the tertiary carbon at C9. Finally, deprotonation leads to the formation of a new double bond between C7 and C8, leading to favorable aromatization of the A-ring that results in the formation of **4**. The mechanism is supported by the observation of the nonenzymatic conversion of **9** into **4** under acidic conditions (Figure S12).

In conclusion, we have identified three enzymes involved in chartreusin biosynthesis that catalyze two mechanistically distinct dehydration reactions. We propose that the 9,10-dehydration reaction by ChaX and ChaU proceeds via a carbanion intermediate, while the mechanism of 7,8-dehydration by ChaJ includes a canonical carbocation intermediate. It is interesting to note that also from an evolutionary perspective,^29^ the two reactions appear to have arisen differently via classical subfunctionalization and neofunctionalization. Ancestral chartreusin pathways may have contained two-component 1-hydroxylase systems that are found on numerous anthracycline pathways.^19^ We show that ChaX has retained the original quinone reduction function, but the subfunctionalization of ChaU has led to the loss of 1-hydroxylation and gain of 9,10-dehydration activity without a gene duplication event (Figure 1F). The difference in enzyme function is presumably mediated via stabilization of different anthracycline resonance forms that lead to carbanion formation at either C1 or C11, but the verification of this hypothesis requires completion of structural studies of ChaU currently in progress in our laboratory. In contrast, the emergence of 7,8-dehydration activity of ChaJ may have arisen differently via neofunctionalization through gene duplication of an ancient fourth ring cyclase ChaK and gain of a novel function through mutations (Figure 1F).

## Supplementary Material



## Notes

Additional biochemical assays, experimental details, materials, and methods. NMR and HRESI-MS spectra for all compounds (PDF)
