# Supplementary material for: Three-Enzyme Cascade Catalyzes Conversion of Auramycinone to Resomycin in Chartreusin Biosynthesis
Source: ACS Chem Biol. 2025 Jul 2;20(7):1457–63. doi: 10.1021/acschembio.5c00205 (PMC12281474; doi:10.1021/acschembio.5c00205)
Supplement: Supplementary file 1 [file cb5c00205_si_001.pdf]

Supporting information for:

Three-enzyme cascade catalyzes conversion of auramycinone to resomycin in chartreusin biosynthesis

Magdalena Niemczura,[‡,1] Aleksi Nuutila,[‡,1] Rongbin Wang,[1] Katariina Rauhanen,[1] S. Eric Nybo[2] and Mikko Metsä-Ketelä[1,*]

[1] *Department of Life Technologies, University of Turku, FIN-20014 Turku, Finland;*
[2] *Department of Pharmaceutical Sciences, College of Pharmacy, Ferris State University, Big Rapids, Michigan 49307, United States;*

* Correspondence: mianme@utu.fi

‡ equal contribution

Experimental procedures 3
General DNA-techniques and microbial cultivations 3
Protein sequence alignment. 4
Protein production and purification. 4
Enzyme assays. 4
Analysis of compounds. 4
Production of 9 for NMR. 5
Production of 4, 6 and 7 for NMR. 5
NMR analysis of 4, 6, 7 and 9 5
Physicochemical Properties of Compounds: 6
Supplementary tables: 6
Table S1. Plasmids and strains: 6
Table S2. Plasmid sequences: 7
Table S3. ^{1}H NMR (500 MHz) data for 4, 6, 7 and 9. 10
Table S4. ^{13}C NMR (125 MHz) data for 4, 7 and 9. 10
Supplementary figures: 11
Figure S1. Protein sequence alignment 11
Figure S2. SDS-PAGE. 12
Figure S3. Main HMBC, COSY and NOESY correlations for 6, 7, 9 and 4. 12
Figure S4. (−)-HRESI-MS of 9. 13
Figure S5. ^{1}H NMR spectrum of 9 in $CDCl_3$, 500 MHz. 14
Figure S6. ^{13}C NMR spectrum of 9 in $CDCl_3$ 15
Figure S7. ^{1}H-^{1}H COSY spectrum of 9 in $CDCl_3$ 16
Figure S8. HSQC spectrum of 9 in $CDCl_3$ 17
Figure S9. HMBC spectrum of 9 in $CDCl_3$ 18
Figure S10. ^{1}H NMR spectrum of 6 in $CDCl_3$, 500 MHz 19
Figure S11. NAD(P)H dependency of KstA16+ChaU reaction 19
Figure S12. Chemical conversion of 9,10-dehydroauramycinone (9) into resomycin C (4). 20
Figure S13. (−)-HRESI-MS of 4. 20
Figure S14. ^{1}H spectrum of 4 in $CDCl_3$, 500 MHz 21
Figure S15. ^{13}C spectrum of 4 in $CDCl_3$ 22
Figure S16. ^{1}H-^{1}H COSY spectrum of 4 in $CDCl_3$ 23
Figure S17. HMBC spectrum of 4 in $CDCl_3$ 24
Figure S18. HSQC spectrum of 4 in $CDCl_3$ 25

Figure S19. (−)-HRESI-MS of 6. 26
Figure S20. (−)-HRESI-MS of 7. 27
Figure S21. ^{1}H spectrum of 7 in $CDCl_3$, 500 MHz 28
Figure S22. ^{13}C spectrum of 7 in $CDCl_3$ 29
Figure S23. ^{1}H-^{1}H COSY spectrum of 7 in $CDCl_3$ 29
Figure S24. HSQC spectrum of 7 in $CDCl_3$ 30
Figure S25. HMBC spectrum of 7 in $CDCl_3$ 30
Figure S26. ^{1}H - ^{1}H NOESY spectrum of 7 in $CDCl_3$ 31
Figure S27. Reactions with the shunt product 7-deoxyauramycinone (7). 31
Figure S28. Peroxide formation in auramycinone (6) reactions with KstA16 and ChaU. 32
References 32

Experimental procedures:

General DNA-techniques and microbial cultivations. All the genetic parts were synthesized (GENEWIZ from Azenta Life Science, South Plainfield, NJ) as standard BioBrick parts with an appropriate BioBrick prefix (5 ′ - GAATTCGCGGCCGCTTCTAGAG-3′) and suffix (5′-TACTAGTAGCGGCCGCTGCAG-3′). Additionally in front of each fragment a ribosome binding site (BBa_B0034) was added within the BioBrick prefix 5′-GAATTCGCGGCCGCTTCTAGAG<u>AAAGAGGAGAAA</u>- TACTAG (underlined). BioBrick parts were cloned using BioBrick restriction enzymes, *EcoRI*, *XbaI*, *SpeI* and *PstI*. High copy number cloning vectors pUC57 and pSBIC3 were used for routine cloning and fragment amplification. Restriction enzymes and the T4 DNA ligase used for cloning were purchased from Fisher Scientific (Fisher Scientific, Waltham, MA).

E. coli TOP10 (Fisher Scientific, Waltham, MA) strain was used for the routine cloning of the BioBrick parts. Chemically competent cells were prepared according to standard procedures. *E. coli* TOP10 were routinely grown on LB agar plates and LB liquid media with an appropriate selection. Media were supplemented as necessary with antibiotics at following concentrations: 100 µg/mL ampicillin, 50 µg/mL apramycin. *E. coli* ET12567/pUZ8002 strain was used as a shuttle strain for the conjugation of the shuttle vector pENTG3 carrying the constructs of interest into *Streptomyces* strain. *S. coelicolor* M1152Δ*matAB* was used for the heterologous expression of the constructs. *S. coelicolor* M1152Δ*matAB* and all derivative strains were routinely grown on solid MS (mannitol-soy flour) agar plates. Spore conjugation was performed according to the standard protocol and the final bacterial suspension was streaked onto a solid MS agar plate supplemented with 10 mM $MgCl_2$ and incubated in +30°C. After 24 h the plates were overlaid with 50 µg/mL apramycin, 10 µg/mL viomycin and 25 µg/mL nalidixic acid and incubated for 72 h at +30°C. After three days of incubation in +30°C six exconjugants were streaked onto a secondary MS plate containing 50 µg/mL apramycin, 10 µg/mL viomycin and 25 µg/mL nalidixic acid and incubated at +30°C for another 3 days until the appearance of bacterial growth.

The vegetative mycelia were scraped from the solid MS agar plates and suspended in liquid TSB (tryptone soy broth) media. Seed cultures were cultured with appropriate antibiotic selection for 3 days in +30°C at 300rpm. 100 µL of the seed culture was inoculated into in 50 mL of SG-TES (10 g soytone, 20 g glucose, 5 g

yeast extract, 5,73 g TES buffer, 1 mg cobalt chloride per one liter) liquid media for 7 days in +30°C at 300rpm in the presence of LXA1180 resin. Strain cultivations were carried out in triplicates.

Protein sequence alignment. To align the cyclase-like enzymes of interest, the amino acid sequences were downloaded from PDB and NCBI databases and aligned with MUSCLE and ClustalO alignment using SeaView5 software package. PyMol was used for structural alignment of published structures from PDB and AlphaFold 3.0 models, which revealed that the MUSCLE alignment was in better agreement with protein structural alignment. A phylogenetic tree was constructed for the MUSCLE alignment using PhyML+LG model in SeaView 5. The MUSCLE alignment file was then uploaded to ESPript 3.0 web server to make an annotated alignment file, in which secondary structure information was fetched from SnoaL2 (PDB: 8R2E) for the top row, and from SnoaL (PDB: 1SJW) for the bottom row (Figure SI1).

Protein production and purification. The *chaU*, *chaJ* and *chaX* genes were cloned into pBHBΔ plasmid using *HindIII* and *NcoI* restriction sites, and transformed using heat-shock method into *E. coli* TOP10 for protein expression. The N-terminal His-tagged proteins were expressed in *E. coli* TOP10 cells in 500 ml aliquots of 2×TY media. The protein expression was induced by adding 0.2 g/L L-arabinose. Induced cells were incubated 250 rpm o/n at RT. Cells were collected through centrifugation, lysed by sonication after 1 h incubation with 0.1 mg/mL lysozyme. Cell debris was removed by 30 min centrifugation at 60 000 × g, and His-tagged protein was purified from the supernatant in a single affinity chromatography step using TALON superflow (Cytiva) protein purification resin according to manufacturer's instructions. 50 mM TRIS buffer system (10 % glycerol and 0.5 M NaCl) was used with 25 mM imidazole for washing and 500 mM imidazole for elution. Imidazole was removed using PD-10 desalting column, after which proteins were concentrated and the glycerol concentration was increased to 50% for storage.

Enzyme assays. Enzyme reactions were carried out in 200 μL of reaction buffer (100 mM phosphate buffer pH 7.5). An aliquot of 4 μL **6** in MeOH was added to the reaction mixture with 5 μM KstA16, 20 μM ChaU, 5.6 μM ChaJ, and 250 μM NADH. Reactions were carried out at 21°C for 30 min.

For the experiments with ChaX, both the TOP10/pBHBΔ-ChaX and TOP10 were cultivated as previously described and the protein expression was carried out as described above. After the production incubation, cells were collected through centrifugation and lysed as described above. The cell lysate was directly used in the enzyme assays to prevent precipitation of ChaX. To maintain high NADH concentration despite the NADH consuming cellular enzymes present in the lysate, 0.5 mM NADH and NADH regeneration system consisting of 30 mM D-glucose and 10 U/ml glucose dehydrogenase (Sigma-Aldrich) were added to the reaction mixture.

Peroxide formation assay. End-point measurement of peroxide formation as a function of time was measured with Pierce™ Quantitative Peroxide Assay Kit (Thermo Scientific) by adding 20 μL of reaction mixture to 200 μL of the Working Reagent according to manufacturer's Aqueous-Compatible Procedure instructions. The peroxide formation was measured at different timepoints (1, 2, 3, 5, 7, 10, 13, 17 and 22 min) for reactions of **6** with KstA16, and KstA16+ChaU to compare the effect of ChaU for peroxide formation. 5 μM KstA16, 20 μM ChaU and 250 μM NADH was used in the reaction mixtures in reaction buffer (100 mM phosphate buffer pH 7.5).

Analysis of compounds. The compounds from *S. coelicolor* M1152Δ*matAB* cultures were extracted from LXA1180 resin using acidic chloroform (1% of 37% HCl in chloroform), dried using a vacuum concentrator (Fisher Scientific) and suspended in 100 μL of DMSO for UHPLC analysis. Samples were analyzed on UHPLC (Shimadzu) with a DAD detector, using a Kinetex C18 1,7 μm 100Å 100×2,1 mm column as

stationary phase. A method used was a 20 min gradient from 10% to 90% (1-15 min), 100% (15-17 min) and 10% (17-20 min). Mobile phase: buffer A was water supplemented with 0.1% TFA; buffer B was acetonitrile supplemented with 0,1% TFA. LCMS analysis was performed using an Agilent 1290 Infinity LC and Agilent 6460 Triple Quadrupole Jetstream MS/MS (Agilent Technologies, Santa Clara, CA). 10 µL of sample was injected into the system. Samples were analyzed on Kinetex C18 1,7 µm 100Å 100×2,1 mm (Phenomenex, Torrance, CA) column using a 20 min gradient method from 10% to 90% (1-15 min), 100% (15-17 min) and 10% (17-20 min). Mobile phase: buffer A was 100% acetonitrile; buffer B was MQ water. Samples were measured in both positive and negative ionization mode with the DAD focused on UV-Vis absorbance of 430 nm and 450 nm. Sample analysis was conducted in Mestrelab Research Analytical Chemistry Software (Mestrelab, Santiago de Compostela, Spain).

The enzyme reaction samples were extracted twice with 1:1 volume of chloroform, dried under reduced pressure and resolubilized in MeOH. The resolubilized enzyme reaction samples were analyzed on UHPLC (Shimadzu) with a DAD detector, using a Kinetex column (2.6 µm C18 100 Å, LC column 100 mm × 4.6 mm, Phenomenex). Samples were analyzed using a 29 min gradient program (0% to 60% solvent B (2-20 min), 100% solvent B (20-24 min) and 0% solvent B (24-29 min), mobile phase: solvent A was 15% acetonitrile in water supplemented with 0.1% HCOOH, and the solvent B was 100% acetonitrile).

Production of 9 for NMR. A large-scale enzyme reaction with KstA16, ChaU and NADPH with **6** as substrate was performed to produce **9** for structure elucidation. The product was extracted from the reaction mixture with repeated chloroform extractions, after which it was dried under reduced pressure using a rotary evaporator, resolubilized in MeOH and further purified using preparative scale HPLC (Agilent Technologies, Santa Clara, CA) using Kinetex 5 µm Phenyl-Hexyl 250×21.2 mm (Phenomenex, Torrance, CA, USA) column with a gradient run from 15% acetonitrile with 0.1% HCOOH to 100% acetonitrile. Collected pure fractions were pooled, dried using a rotary evaporator and nitrogen flow. After the NMR measurement in $CDCl_3$, a drop of CD_3OD was added to the NMR-sample to remove the signal of exchangeable protons to confirm the determination of 7-OH signal (2.23 ppm), and ^1H spectrum was remeasured. As a consequence, the splitting pattern of 7H signal lost the 4.9 Hz ^3J coupling to the 7-OH proton.

Production of 4, 6 and 7 for NMR. The appropriate strains were inoculated into 50 mL of SG media and cultured for 5 days in 30°C at 300rpm. 100µl of the preculture was inoculated into 10 250ml flasks containing 50ml of SG-TES media and cultured for 7 days in 30°C at 300rpm. The cultures were pooled and extracted with chloroform with 1% HCl, dried in a rotary evaporator (Buchi, Flavil, Switzerland), and suspended in DMSO. The compounds in DMSO were then purified using preparative HPLC (Agilent Technologies, Santa Clara, CA) using Kinetex 5 µm EVO C18 250×21.1 mm (Phenomenex, Torrance, CA, USA) column. Each of the pure compounds were later dried in rotary evaporator and weighed.

NMR analysis of 4, 6, 7 and 9. For NMR analysis, all the dried samples were dissolved in deuterated chloroform with 0.03% v/v TMS. NMR analysis of the samples was conducted using Bruker Avance Neo 500 with Oxford 500 MHz non-shielded magnet and BB/1H Smartprobe. All NMR spectra were analyzed using TopSpin 4.1.3 version (Bruker BioSpin Corporation, Billerica, MA), and *J*-coupling constants were simulated and extracted using ChemAdder (Spin Discoveries Ltd.).

Physicochemical Properties of Compounds:

Auramycinone (**6**): $C_{21}H_{18}O_8$, yellow solid, HPLC R_t (method 1, mobile phase: A:MQ+0,1%TFA; B: ACN+0,1%TFA, Kinetex C18 1,7µm 100Å 100x2,1mm) = 9,14min, UV-vis = 225nm, 258nm, 430nm, m/z=398,10

Resomycin C (**4**): $C_{21}H_{14}O_6$, orange solid, HPLC R_t (method 1, mobile phase: A:MQ+0,1%TFA; B: ACN+0,1%TFA, Kinetex C18 1,7µm 100Å 100x2,1mm) = 13,265min, UV-vis = 241nm, 259nm, 287nm (sh), 448nm, m/z=362,3

9,10-dehydroauramycinone (**9**): $C_{21}H_{16}O_7$, yellow solid, HPLC R_t (method 1, mobile phase: A:MQ+0,1%TFA; B: ACN+0,1%TFA, Kinetex C18 1,7µm 100Å 100x2,1mm) = 9,868min, UV-vis = 222nm, 259nm, 286nm, 443nm, m/z=380,35

7-deoxyauramycinone (**7**): $C_{21}H_{18}O_7$, dark yellow solid, HPLC R_t (method 1, mobile phase: A:MQ+0,1%TFA; B: ACN+0,1%TFA, Kinetex C18 1,7µm 100Å 100x2,1mm) = 10,242min, UV-vis = 228nm, 259nm, 432nm, m/z=382,08

Supplementary tables:

Table S1. Plasmids and strains:

Plasmid/Strain	***Source***
pEN10003	(Wang et al., 2022)
pAURA2	This study
pENTG3_SP44_KstA16_gapdhP_chaU	This study
pENTG3_SP44_KstA16_gapdhP_chaU_gapdhP_chaJ	This study
pENTG3_gapdhP_chaX_gapdhP_chaU_gapdhP_chaJ	This study
pENTG3_gapdhP_chaX	This study
Streptomyces coelicolor M1152ΔmatAB	
Escherichia coli TOP10	
Escherichia coli ET12567 / pUZ8002	
M1152ΔmatAB_pAURA2_pENTG3_SP44_KstA16_gapdhP_chaU	This study
M1152ΔmatAB_pAURA2_pENTG3_SP44_KstA16_gapdhP_chaU_gapdhP _chaJ	This study
M1152ΔmatAB_pAURA2_pENTG3_gapdhP_chaX	This study
M1152ΔmatAB_pAURA2_pENTG3_gapshP_chaX_gapdhP_chaU_gapdhP _chaJ	This study

Table S2. Plasmid sequences:

1. **pOSV821-AURA2**

>pAURA2

AAAAAAAAAAGCCCCGCGATTGCGGGGCTTTTTTTTTATGCATGCGGCCGCTGCTAGAGTGTTCACATTCGAACCGTCTCTGCTTTGACAACATGCTGTGCGGTGTTGTAAAGTCCAGCTGAAGTAC
GTCTGAGCGTGATACCCGCTCACTGAAGATGGCCCGGTAGGGCCGAAACGTACCTCTACAAATAATTTTGTTTAATACTAGAGGAAGGGCATCTTCGACGAATGAAGGAATCCATCAACCGTCGCGT
GGTCATCACCGGAATAGGGATCGTCGCGCCCGATGCCACCGGGGTGAAACCGTTCTGGGATCTGCTGACGGCCGGTCGCACCGCGACCCGGACCATCACCGCCTTCGATCCCTCTCCGTTCCGTTCCC
GCATCGCCGCGGAATGCGATTTCGACCCGCTTGCCGAAGGGCTGACCCCCCAGCAGATCCGGCGTATGGACCGGGCCACGCAGTTCGCGGTCGTCAGCGCCCGGGAAAGCCTGGAGGACAGCGGAC
TCGACCTCGGCGCCCTGGACGCCTCCCGCACCGGCGTGGTCGTCGGCAGCGCGGTCGGCTGCACCACGAGCCTGGAAGAGGAGTACGCGGTCGTCAGCGACAGCGGCCGGAACTGGCTGGTCGACG
ACGGCTACGCCGTACCGCACCTATTCGACTACTTCGTGCCCAGCTCCATCGCCGCCGAGGTGGCACACGACCGCATCGGCGCGGAGGGCCCCGTCAGCCTCGTGTCGACCGGGTGCACCTCGGGCCT
GGACGCCGTGGGCCGCGCGGCCGACCTGATCGCCGAGGGAGCGGCGGATGTGATGCTGGCCGGTGCGACCGAGGCGCCCATCTCCCCCATCACCGTGGCGTGCTTCGATGCCATCAAGGCGACCAC
CCCCCGCAACGACACGCCCGCCGAGGCGTCCCGTCCGTTCGACCGCACCAGGAACGGGTTCGTACTCGGCGAGGGCGCTGCCGTGTTCGTCCTGGAGGAGTTCGAACACGCGCGCCGCCGGGGCGC
GCTCGTGTACGCGGAGATCGCCGGGTTCGCCACTCGCTGCAACGCCTTCCACATGACCGGTCTGCGCCCGGACGGGCGGGAGATGGCGGAGGCCATCGGGGTGGCGCTCGCCCAGGCGGGCAAGGC
GCCGGCTGACGTCGACTACGTCAACGCCCACGGTTCCGGCACCCGGCAGAATGACCGTCACGAGACGGCGGCCTTCAAGCGCAGTCTCGGCGACCACGCCTACCGGGTCCCGGTCAGCAGCATCAA
ATCCATGATCGGGCACTCGCTGGGCGCGATCGGCTCCCTGGAGATCGCCGCCTCCGTGCTGGCCATCACACACGACGTGGTGCCGCCCACCGCCAATCTGCACGAGCCGGATCCCGAGTGCGATCTG
GACTACGTGCCGCTGCGGGCGCGTGCGTGCCCGGTGGACACGGTGCTCACGGTGGGCAGCGGGTTCGGCGGTTTCCAGAGCGCCATGGTGCTGTGCGGTCCGGGCTCGCGGGGAAGGTCGGCCGCGT
GACGGCCGCCGTGGTGGTGACCGGTCTCGGCGTCGTCGCCCCCACCGGTCTCGGGGTGCGGGAGCACTGGTCGAGTACGGTCCGGGGGGCGTCGGCGATCGGACCGGTCACCCGGTTCGACGCCGG
CCGGTACCCCAGCAAACTGGCCGGAGAGGTGCCCGGTTTCGTCCCGGAGGACCATCTGCCCAGCCGGCTGATGCCGCAGACGGACCATATGACGCGCCTGGCGCTCGTCGCGGCGGACTGGGCCTTC
CAGGACGCCGCCGTGGACCCGTCGAAGCTGCCGGAGTACGGCGTCGGCGTGGTCACCGCGAGTTCGGCGGGGGGGTTCGAGTTCGGCCACCGCGAGCTCCAGAACCTGTGGAGCCTGGGCCCGCAG
TACGTCAGCGCGTATCAGTCGTTCGCATGGTTCTATGCCGTGAACACCGGTCAGGTGTCCATCCGGCACGGGCTGCGCGGCCCGGGCGGGGTGCTGGTGACGGAACAGGCGGGCGGCCTGGACGCCC
TTGGGCAGGCCCGGCGGCAGTTGCGGCGCGGACTGCCGATGGTGGTCGCGGGAGCCGTTGACGGCTCGCCCTGCCCCTGGGGCTGGGTGGCGCAGCTCAGCTCGGGCGGCCTCAGCACGTCGGACG
ACCCGCGCCGGGCCTATCTGCCGTTCGACGCCGCAGCCGGCGGACACGTGCCGGGAGAGGGCGGCGCCCTGCTCGTCCTGGAGAGCGACGAGTCGGCCCGGGCGCGCGGGGTGACGCGGTGGTACG
GGCGCATCGATGGGTACGCCGCCACATTCGACCCCCCGCCCGGTTCGGGGCGCCCGCCGAACCTGCTGCGGGCCGCGCAGGCGGCACTGGACGACGCGGAGGTCGGACCCGAGGCGGTCGACGTGG
TGTTCGCGGACGCGTCCGGCACCCCGGACGAGGACGCGGCGGAGGCCGACGCGGTGCGGCGCCTGTTCGGACCGTACGGCGTTCCGGTGACGGCGCCGAAGACCATGACCGGCCGCCTCAGCGCGG
GCGGCGCGGCCCTCGACGTGGCGACGGCGCTGCTGGCGCTGCGCGAGGGCGTCGTCCCGCCGACGGTCAACGTCTCCCGGCCGCGGCCGGAGTACGAGCTGGACCTGGTGCTCGCCCCCCGGCGCA
CGCCCCTGGCCAGGGCCCTGGTGCTCGCGCGGGGCCGGGGCGGGTTCAATGCGGCGATGGTCGTGGCGGGGCCGCGCGCGGAGACACGGTGAAGCGGCCCGGCGCAGCCGGAGCCGCGGTAAGAG
GCCACGGAAGAGAGAGGGATGCGACGGTGAAGCAGCAGCTGACGACGGAACGGCTCATGGAGATCATGCGGGAGTGCGCGGGCTACGGTGAGGACGTCGACGCTCTGGGCGACACGGACGGCGCC
GACTTCGCCGCACTCGGCTACGACTCGCTGGCGCTCCTGGAAACGGCCGGCCGGCTCGAGCGCGAGTTCGGCATCCAGCTCGGTGACGAGGTGGTCGCCGACGCCAGGACGCCTGCCGAGCTGACCG
CCCTGGTCAACCGGACGGTGGCCGAGGCGGCCTGATACTAGAGTGTTCACATTCGAACCGTCTCTGCTTTGACAACATGCTGTGCGGTGTTGTAAAGTCGGGTGAAAGTCCGTAGTGGATGTGTATCC
ACTCTGATGAGTCCGAAAGGACGAAACGGACCTCTACAAATAATTTTGTTTAATACTAGAGGAGGAGCGAGCATGCCGGACACGGACGGGCGGGTCGCCGTCGTCACAGGCGGCACCAGCGGGATA
GGACTCGCGGTGGTGAAGGCACTGGCGCGACAGGATATGCGTGTCGTCCTGTGTGCCCGGAGCCAGGACGCCGTCGACAGCACCGTGGAGGAGCTGCGCGACGAGAAGCTGGAGGTGGACGGGCG
CGCCTGCGACGTGCGTTCCACGGACGACGTACGGGAGTTCATCGGCTTCGCCACGCGGCGCTTCGGCGGCGTGGACGTGCTGGTCAACAACGCCGGCCGCAACGGCGGAGGCCCGACCGCGCGGAT
AACCGACGAGCTGTGGCTCGACGTCATCGAGACCAACCTCACCAGCGTCTTCCGCGTCACCCGCGAGGTGCTGACCACCGGAGGGATGCTCGCCGCCGGACGCGGCAGGATCATCAACATCGCCTCC
ACCGGCGGCAAACAGGGTGTCGTCCTCGGTGCCCCGTACTCGGCCTCCAAGCACGGCGTCGTCGGCTTCACCAAGGCACTGGGCCTGGAGCTGGCCAAGAGCGGCATCACCGTCAACGCCGTCTGCC
CGGGCTATGTCGAGACTCCCATGGCCGAGCGGGTGCGTCGCGGCTACGCCGAGGTCTGGGACACCTCCGAGGAGGCGGTGCTGGAGCGCTTCCAGGCGAAGATCCCGCTCGGCCGCTACGCGCAGC
CCGAGGAGGTCGCCGGCCTCGTCACCTACCTGACCACGCCCACCGCCGACTCCATCACCGCGCAGGCGCTCAACGTCTGCGGCGGCCTGGGAAACTACTGATACTAGAGGGACGGAGGACCATGAC
AGCCGGCGACACCACCCTCACCACCCACCGCACCGAACACCGCGTCACCGTCTCGGCACCCGCGCGGCGCGTCTTCGACCTGGTCGCGGACATCACAGGCTGGCCCCACACCTTCCCCCCGACCGTG
CACGCGGAGTACGCCGAGCGCGGGGCGAGTGAGGAACGCATCCGCATCTGGGCCACGGCCAACGGCGAGGTCAAGGCCTGGACCTCGCACCGCTCGCTGGACCGGGAAGGGCTGCGCGTCCGCTTC
CGGCAGGAGAAGTCCCAGCACCCCGTGGCCGGCATGGGCGGCGAGTGGATCATCCGGCCGGTGGACGGCGAGCGGAGCGAGGTGATCCTCACCCACGACTTCCAGGCCGTGGAGGACGACCCGGC
CCACGTGGACTGGATCCACCGTGCCGTGGACCGCAACAGCGGCGCCGAACTCGCGGCGCTGAAAGCCGCGGCGGAACGCGCGGACGGCGCGGACGGCACCCTGTTCACCTTCGCCGACGAGGTCAC
CGTCACCGGTGCCCCCGGTGACGTCTACGACTACCTCAACGAGGCCCGGCGCTGGCAGGAGCGCCTCCCGCACGTCGCCGCGGTGAGCCTCGACGAGCCCGCCCCCGGAGTGCAGCGGCTCGGCATG
GACACCAAGGGACCCGACGGAACCGTGCACACCACCGAGTCGGTCCGCATCTGCTTCCCCCAGGACCGCATCGTCTACAAGCAGTTCACGATGCCACCGCTGATGGCCGTCCATACCGGCACATGGC
GGATCGCACCCGCCGCGGACGGCCACGGCACGACCGTCACCTCCCTCCACACCGTCGTGGTCGACGACAGCGCCGTCACCACCGTCCTGGGCCCGGACGCCACCCTCGCCGACGCCCGCCGCTTCCT
GCGTGACGCCCTCGGCCGCAACAGCCGTACCACCCTCGGCTTCGCCAAGGAATACGCCGAGGAGCGCGCGCGGCGGGCCTGATACTAGAGGTGGATGCCGCGGAGCCACTGGTAACCACGCAGCGA
GAGCAGCGCACGCCCCAGCTCGCTGTCCGTCAGTTTCATCACCATGACGGCAGCGTGGGGCGCGCCGCTATACCCTCCCTGGATTCCCGCTCGGCCGGGACGGCGCCGGTGCGGCGCCGGCCGTCGC
GTGCGGTGCCCGCCCGTCGAACCAGCTTCCAGGCCACGTCAAGAGGGGCGTGTCAGCGTCGTACCCGCGCTGCGTCAGCCGAGCCCGTCTACGAACCCGGAGGTGCGCGTGCGGCTCATCGACCTTT
CGTCACCGGTGGATTCCTCACAGTACGAGCCCGACCCGGTGGTGCACGACGTCCTCACGCCCCGGCAGGGCGCGGAACACATGTGCGCGGAGATGCGCGAGCACTTCGGTGTCGAGTTCTCTCCGGA
CGAACTGCCGGACGGCGAGTTCCTGTCACTGGACAGGATCACGCTGACCACGCACACCGGCACGCACGTCGACGCGCCGTCGCACTATGGTTCGCGCGCGCTGTACGGGGACGGCGTCCCCCGCCAC
ATCGACCAGATGCCGCTGGAGTGGTTCTTCGGGCGCGGGGTGGTGCTGGACCTCACCGACGCTCCGACGGGCACCGTGAGCGCCGCGCGCCTGGAGAAGGAACTGGCCCGGACCGGGTGCGCGTTG
CGGCCCGGGGACATCGTGCTGCTGCACACCGGTGCCCAGCGGCACGCGGGCACCCCGCGCTACTTCACGGACTTCGCCGGTCTCGACGGGCCCGCGGTACGGATGCTTCTCGACCACGGGGTCCGGG
TGATCGGGACCGATGCCTTCAGCCTGGACGCGCCCTTCGGCCACATCATCGACCGGTACCGCGCGACCGGCGACCGATCGGTGCTGTGGCCCGCGCACGTGGTGGGCCGGGAGCGGGAGTACTGCCA
GATCGAGCGGCTGGCGAACCTCGACCGCCTGCCGGTCTCCTTCGGGTTCCGCGTCTGCTGCTTCCCGGTCAAGGTCGCCGGCGCCGGCGCCGGGTGGACCCGGGCGGTCGCCCTGGTGGACGAGGAC
TGATACTAGAGAGGGAGTCGTGGACACATGCCGACACGAGTGAACGATGGCGTCGACGCGGACGAGGTCACCTTCGTCAACAGGTTCACCGTCCACGGGGCGCCCGCGGAGTTCGAGAGCGTCTTC
GCACGGACCGCGGCCTTCTTCGCGCGGCAGCCCGGGTTCGTCCGGCATACGCTGCTGCGCGAGCGGGACAAGGACAACAGCTATGTGAACATCGCCGTCTGGACCGACCACGACGCCTTCCGGCGG
GCCCTGGCCCAGCCCGGGTTCCTGCCGCACGCCACCGCGTTGCGGGCCCTGAGCACCAGCGAACACGGGCTGTTCACGGCGCGCCAGACACTGCCGGAAGGCGGCGATACGACCGGCTCCGGACAT
CGCTGATACTAGAGTGTTCACATTCGAACCGTCTCTGCTTTGACAACATGCTGTGCGGTGTTGTAAAGTCTGGTGTAAGCTGTCACCGGATGTGCTTTCCGGTCTGATGAGTCCGTGAGGACGAAACA
GCCTCTACAAATAATTTTGTTTAATACTAGAGAGAAGGAGCAGGCGTGAGTGAGCAGATAGCGGCCGTTCGGCGCATGGTCGAGGCGTACAACACCGGAAAAACGGATGATGTGGCCGACTACATC
CATCCGGAATACATGAATCCGGGAACCCTGGAGTTCACCTCGCTGCGTGGCCCGGAACTCTTCGCGATCAATGTCGCGTGGGTCAAGAAGACGTTCTCCGAGGAGGCGCGGCTGGAAGAGGTCGGCA
TCGAGGAGCGGGCCGACTGGGTGCGCGCCCGGCTGGTGCTCTACGGCCGGCACGTCGGTGAGATGGTCGGCATGGCGCCGACCGGACGGCTGTTCTCCGGCGAGCAGATCCATCTCCTGCACTTCGT
CGACGGGAAGATCCACCACCACCGCGACTGGCCCGACTACCAGGGCACCTACCGCCAGCTCGGCGAGCCGTGGCCGGAGACCGAGCACCGCCGTCCCTGATACTAGAGGAGAGAGCGGGAGAGCC
AGTGGACACGTCCACCTACAAATCCGAAGTGGCGGCCGCCTTCAACAGCGCCGCGGCGACCTACGACCGGCTCGGCGTGGAGTTCTTCACCCCGATGGGCCGGCGTCTGGTGGAACGCGCGGCACC
GGCCGCCGGCGCCCGGGTCCTCGACGTCGGCTGCGGGCGGGGCGCGTGCGTCTTCCCGGCTGCCGAACGCGTGGGGCCCGAGGGCCGTGTCGTGGGCATCGACGTCGCCGAGGCGATGATCGAGGA
GGCCACGAAGGAGGCGGCGCTGCGCGACGCCGCCATGGTCGAGCTGCGGGTGATGGACGGGGAGCACCCCGACTTCGCGCCGCACTCCTTCGACGTGGTCCTGGGCAGCTACAGCGTCATCTTCCTG
CCCGACGCCCCGACGGCGCTGGCCCGTTACGCGCCGCTGCTCTCCCCCGGGGGCCGACTCGCCTTCACCAGCCCGGTGTTCAGCGACGACACCTTCCCCTTCCTGCCCCCGGTGTTCACCGAACTGAT
CCCGCGCGAGCTGCTGCTCGACCTGCCGCCGCAGTGGCAGCCCGAACAGCTCCAGCAGCGCTTCAACAGCTGGCTCGCGGACCCGCGGGACCTCACCCGCACACTGGAGCGGTGCGGCTTCCAGGA
GGTGACGGTCGTCGACGAACCCGTGACTCTGGTGGCCGAATCGGGCCTGGCCTGGGTGGACTGGTCGCACACCCAGGGGATGCGCCTGCTCTGGAACCACCTGGCGCAGGACAAACGTCAGCAGCT
GCGGGAGCGGCTGATCACCTCGCTCGACGCGATGCGGGACGGGGACGGCCCGCTCACGATCGACACCCCGGTGCGGTACGTCACCGCGACCGTACGGAACTGATACTAGAGGAGCGGGAGTCGACC
GTGAATCGAGCGGCCCGGCCTACGGTCCGGGGCATGAGCGCCATAGCAGAACCGACAGCGCCGCGGGGCGTGATCGTCACCGGCGGCGGGACCGGGATCGGGCGGGCCACGGCCCACGCCTTCGCC
GACCGCGGCGACCGGGTACTCGTCGTAGGCCGCACCGCCGCGACCCTGGCCGGGACCGCCGAGGGCCACCCCGGCATCAGTGTCCTGACCGCCGATCTCACCGATCCGGACGGTCCGCGCGCCATCA
CCGACGCGGCGCTGGACGCGTTGGGCCGTATCGACGTCCTCGTGAACAACGCGGCCACCGGTGGGTTCGCCGGCCTGGCGGAGACCGAGCCGGAGGCCGCCCGCGAGCAGTTCGACAGCAACCTCC
TGGCCCCGCTGCTGCTCACCCGGCAGACCCTGGACGCCCTGTCGGCCGACGGCGGGGGAACCGTGCTGAACATCGGCTCGGCCGGAGCTCTGGGCCGCCGGGCCTGGCCGCAGAACGGTGTCTACG
GCGCCGCCAAGGCCGGGCTCGACTTCCTCACCCGGACCTGGGCCGTCGAACTGGCACCCCGCGGCATCCGTGTCCTCGGCCTGGCGCCCGGCGTGATCGACACCGGCATCGGTGAACGCTCGGGCAT
GTCACGGGAGGCCTACGCGGGCTTCCTCGGGCAGATCGCCGCCCGCGTCCCGGCCGGCCGGGTCGGCCGGCCCGAGGACATCGCCTGGTGGGCCGTCCAGCTGGCCGACCCGCGCGCCGCTTACGCG
ACCGGAGCGGTGCTCGCGGTCGACGGCGGTCTGTCCCTCACCTGATACTAGTAGCGGCCGCTCTAAAGTTTTGTCGTCTTTCCAGACGTTAGTAAATGAATTTTCTGTATGAGGTTTTGCTAAACAACT
TTCAACAGTTTCAGCGGAGTGAGAATAGAAAGGAACAACTAAAGGAATTGCGAATAATAATTTTTTCACGTTGAAAATCTCCAAAAAAAAAGGGCTCCAAAAGGAGCCTTTAATTGTATCGGTTTATC
AGCTTGCTTTCGAGGTGAATTTCTTAAACAGCTTGATACCGATAGTTGCGCCGACAATGACAACAACCATCGCCCACGCATAACCGATATATTCGGTCGCTGAGGCTTGCAGGGAGTCAAAGGCCGC
TTTTGCGGGATTAAGCAGGCTTCCCGGGTGTCTCGCTACGCCGCTACGTCTTCCGTGCCGTCCTGGGCGTCGTCTTCGTCGTCGTCGGTCGGCGGCTTCGCCCACGTGATCGAAGCGCGCTTCTCGATG
GGCGTTCCCTGCCCCCTGCCCGTAGTCGACTTCGTGACAACGATCTTGTCTACGAAGAGCCCGACGAACACGCGCTTGTCGTCTACTGACGCGCGCCCCCACCACGACTTAGGGCCGGTCGGGTCAGC
GTCGGCGTCTTCGGGGAACCATTGGTCAAGGGGAAGCTTCGGGGCTTCGGCGGCTTCAAGTTCGGCAAGCCGCTCTTCCGCCCCTTGCTGCCGGAGCGTCAGCGCTGCCTGTTGCTTCCGGAAGTGCT
TCCTGCCAACGGGTCCGTCGTACGCGCCTGCCGCGCGGTCTTCGTACAGCTCTTCAAGGGCGTTCAGGGCGTCGGCGCGCTCCGCAACAAGGTTCGCCCGTTCGCCGCTCTTCTCAGGCGCCTCAGTG
AGCTTGCCGAAGCGTCGGGCGGCTTCCCACAGAAGCGCCAACGTCTCTTCGTCGCCTTCGGCGTGCCTGATCTTGTTGAAGATGCGTTCCGCAACGAACTTGTCGAGTGCCGCCATGCTGACGTTGCA
CGTGCCTTCGTGCTGCCCAGGTGCGGACGGGTCGACCACCTTCCGGCGACGGCAGCGGTAAGAGTCCTTGATCGATTCTTCCCCGCGCTTCGAAGTCATGACGGCGCCACACTCGCAGTACAGCTTGT
CCATGGCGGACAGAATGGCTTGCCCCCGGGAAAGCCCCTTGCCGCGCCCCCTGCCGTCCAACCACGCCTGAAGCTCATACCACTCAGCGGGCTCGATGATCGGTCCGCAATCAAGCTCGACCGGCCG

GAGCGTGATCGGGTCGCGCTGAATGCGGTAACCCTCAATCTTCGTGGTCGGCGTGCCGTCCGGCTTCTTCTTGTAGATCACCTCAGCGGCGAAGCCCGCAATACGCGGGTCCCGAAGGATTCGCATAA
CGGTTGCCGGGTCCCAGGCGCTTGAAGCGGTCTTCTTCCCAATCGTCTCGCCCCGGGTCGGCACGGCGTCAGCGTCCATGCGCTTACAAAGCCCCGTGATGCTGCCCGGGTGAATGGCGGCTTGACTG
CCCGGCTTGAAGGGAAGGTGTTTGTGCGTCTTGATCTCACGCCACCACCACCGGATTACGTCGGGCTCGAACTCGAAGGGTCCGGTAAGGGGAGTGGTCGAGTGCGCAAGCTTGTTGATGACGACAT
TGACCATTCGGCCGTTGCGCGTGATCTCCTTCGTCTCCGAAACAAGCTCGAAGCCGTAAGGCGCCTTCCCGCCGACGTACCCGCCCAATTCGCGCTGAAGGTTCTTCGTGTCGAGAATCTTCGCCGAC
TTCAGCGAAGATTCTTTGTGCGACGCGTCGAGCCGCATAATCAGGTGAATCAGGTCCATGACGTTTCCCTGCCGGAAGACGCCTTCCTGAGTGGAAACAATCGTCACGCCCAGGGCGAGCAATTCCG
AGACAATCGGAATCGCGTCCATGACCTTCAGGCGCGAGAAGCGCGACACGTCATAGACAATGATCATGTTGAGCCGCCCGGCGCGGCATTCGTTCAGGATGCGTTCGAACTCCGGGCGCTCCGCCGT
CCCGAACGCCGACGTGCCCGGCGCTTCGCTGAAATGCCCGACGAACCTGAACCGGCCCCCGTCGCGCTCGACTTCGCGCTGAAGGTCGGCCGCCTTGTCTTCGTTGGCGCTACGCTGTGTCGCTGGGC
TTGCTGCGCTCGAATTTTCGCGCTCGCGCGACTGACGGTCGTAAGCACCCGCGTACGTGTCCACCCCGGTCACAACCCCTTGTGTCATGTCGGCGACCCTACGCCCCCAACTGAGAGAACTCAAAGGT
TACCCCAGTTGGGGCACTACTCCCGAAAACCGCTTCTGACCTGGGAAAACGTGAAGCCCCGGGGCATCCGCTGAGGGTTGCCGCCGGGGCTTCGGTGTGTCCGTCAGTACGGGCCATAGAGGGGCGT
CGTGTAACCCACCCAGGGCGATTGCTCCGGCACGGGGAAGCCGCGCCACGCCTTCGGGACGTCTGGAATCCTGCAGGGAAGTTCCTATTCTCTAGAAAGTATAGGAACTTCGTCCACGACGCCCGTG
ATTTTGTAGCCCTGGCCGACGGCCAGCAGGTAGGCCGACAGGCTCATGCCGGCCGCCGCCGCCTTTTCCTCAATCGCTCTTCGTTCGTCTGGAAGGCAGTACACCTTGATAGGTGGGCTGCCCTTCCT
GGTTGGCTTGGTTTCATCAGCCATCCGCTTGCCCTCATCTGTTACGCCGGCGGTAGCCGGCCAGCCTCGCAGAGCAGGATTCCCGTTGAGCACCGCCAGGTGCGAATAAGGGACAGTGAAGAAGGAA
CACCCGCTCGCGGGTGGGCCTACTTCACCTATCCTGCCCGGCTGACGCCGTTGGATACACCAAGGAAAGTCTACACGAACCCTTTGGCAAAATCCTGTATATCGTGCGAAAAAGGATGGATATACCG
AAAAAATCGCTATAATGACCCCGAAGCAGGGTTATGCAGCGGAAAAGGGTACCACGTCGCGGTGAGTTCAGGCTTTTTCATATCTCATTGCCCCCGGACGAGCGTCTGCTCCGCCATTCGCCGTCCGC
CGTGCCAATCGGATCAGCCGTCCAAATGCGGGATTTTCGTTAGTCGGAGGCCAAACGGCATTGAGCGTCAGCATATCATCAGCGAGCTGAAGAAAGACAATCCCCGATCCGCTCCACGTGTTGCCCC
AGCAATCAGCGCGACCTTGCCCCTCCAACGTCATCTCGTTCTCCGCTCATGAGCTCAGCCAATCGACTGGCGAGCGGCATCGCATTCTTCGCATCCCGCCTCTGGCGGATGCAGGAAGATCAACGGAT
CTCGGCCCAGTTGACCCAGGGCTGTCGCCACAATGTCGCGGGAGCGGATCAACCGAGCAAAGGCATGACCGACTGGACCTTCCTTCTGAAGGCTCTTCTCCTTGAGCCACCTGTCCGCCAAGGCAAA
GCGCTCACAGCAGTGGTCATTCTCGAGATAATCGACGCGTACCAACTTGCCATCCTGAAGAATGGTGCAGTGTCTCGGCACCCCATAGGGAACCTTTGCCATCAACTCGGCAAGATGCAGCGTCGTG
TTGGCATCGTGTCCCACGCCGAGGAGAAGTACCTGCCCATCGAGTTCATGGACACGGGCGACCGGGCTTGCAGGCGAGTGAGGTGGCAGGGGCAATGGATCAGAGATGATCTGCTCTGCCTGTGGCC
CCGCTGCCGCAAAGGCAAATGGATGGGCGCTGCGCTTTACATTTGGCAGGCGCCAGAATGTGTCAGAGACAACTCCAAGGTCCGGTGTAACGGGCGACGTGGCAGGATCGAACGGCTCGTCGTCCA
GACCTGACCACGAGGGCATGACGAGCGTCCCTCCCGGACCCAGCGCAGCACGCAGGGCCTCGATCAGTCCAAGTGGCCCATCTTCGAGGGGCCGGACGCTACGGAAGGAGCTGTGGACCAGCAGCA
CACCGCCGGGGGTAACCCCAAGGTTGAGAAGCTGACCGATGAGCTCGGCTTTTCGCCATTCGTATTGCACGACATTGCACTCCACCGCTGATGACATCAGTCGATCATAGCACGATCAACGGCACTG
TTGCAAATAGTCGGTGGTGATAAACTTATCATCCCCTTTTGCTGATGGAGCTGCACATGAACCGGATCCCCTAGCGGAGTGTATACTGGCTTACTATGTTGGCACTGATGAGGGTGTCAGTGAAGTGC
TTCATGTGGCAGGAGAAAAAAGGCTGCACCGGTGCGTCAGCAGAATATGTGATACAGGATATATTCCGCTTCCTCGCTCACTGACTCGCTACGCTCGGTCGTTCGACTGCGGCGAGCGGAAATGGCT
TACGAACGGGGCGGAGATTTCCTGGAAGATGCCAGGAAGATACTTAACAGGGAAGTGAGAGGGCCGCGGCAAAGCCGTTTTTCCATAGGCTCCGCCCCCCTGACAAGCATCACGAAATCTGACGCT
CAAATCAGTGGTGGCGAAACCCGACAGGACTATAAAGATACCAGGCGTTTCCCCCTGGCGGCTCCCTCGTGCGCTCTCCTGTTCCTGCCTTTCGGTTTACCGGTGTCATTCCGCTGTTATGGCCGCGTT
TGTCTCATTCCACGCCTGACACTCAGTTCCGGGTAGGCAGTTCGCTCCAAGCTGGACTGTATGCACGAACCCCCCGTTCAGTCCGACCGCTGCGCCTTATCCGGTAACTATCGTCTTGAGTCCAACCCG
GAAAGACATGCAAAAGCACCACTGGCAGCAGCCACTGGTAATTGATTTAGAGGAGTTAGTCTTGAAGTCATGCGCCGGTTAAGGCTAAACTGAAAGGACAAGTTTTGGTGACTGCGCTCCTCCAAGC
CAGTTACCTCGGTTCAAAGAGTTGGTAGCTCAGAGAACCTTCGAAAAACCGCCCTGCAAGGCGGTTTTTTCGTTTTCAGAGCAAGAGATTACGCGCAGACCAAAACGATCTCAAGAAGATCATCTTA
TTAATCAGATAAAATATTTCTAGATTTCAGTGCAATTTATCTCTTCAAATGTAGCACCTGAAGTCAGCCCCATACGATATAAGTTGTGAAGTTCCTATTCTCTAGAAAGTATAGGAACTTC

2. pEN10003

>pEN10003

CAGGCTTCCCGGGTGTCTCGCTACGCCGCTACGTCTTCCGTGCCGTCCTGGGCGTCGTCTTCGTCGTCGTCGGTCGGCGGCTTCGCCCACGTGATCGAAGCGCGCTTCTCGATGGGCGTTCCCTGCCCC
CTGCCCGTAGTCGACTTCGTGACAACGATCTTGTCTACGAAGAGCCCGACGAACACGCGCTTGTCGTCTACTGACGCGCGCCCCCACCACGACTTAGGGCCGGTCGGGTCAGCGTCGGCGTCTTCGG
GGAACCATTGGTCAAGGGGAAGCTTCGGGGCTTCGGCGGCTTCAAGTTCGGCAAGCCGCTCTTCCGCCCCTTGCTGCCGGAGCGTCAGCGCTGCCTGTTGCTTCCGGAAGTGCTTCCTGCCAACGGGT
CCGTCGTACGCGCCTGCCGCGCGGTCTTCGTACAGCTCTTCAAGGGCGTTCAGGGCGTCGGCGCGCTCCGCAACAAGGTTCGCCCGTTCGCCGCTCTTCTCAGGCGCCTCAGTGAGCTTGCCGAAGCG
TCGGGCGGCTTCCCACAGAAGCGCCAACGTCTCTTCGTCGCCTTCGGCGTGCCTGATCTTGTTGAAGATGCGTTCCGCAACGAACTTGTCGAGTGCCGCCATGCTGACGTTGCACGTGCCTTCGTGCTG
CCCAGGTGCGGACGGGTCGACCACCTTCCGGCGACGGCAGCGGTAAGAGTCCTTGATCGATTCTTCCCCGCGCTTCGAAGTCATGACGGCGCCACACTCGCAGTACAGCTTGTCCATGGCGGACAGA
ATGGCTTGCCCCCGGGAAAGCCCCTTGCCGCGCCCCCTGCCGTCCAACCACGCCTGAAGCTCATACCACTCAGCGGGCTCGATGATCGGTCCGCAATCAAGCTCGACCGGCCGGAGCGTGATCGGGT
CGCGCTGAATGCGGTAACCCTCAATCTTCGTGGTCGGCGTGCCGTCCGGCTTCTTCTTGTAGATCACCTCAGCGGCGAAGCCCGCAATACGCGGGTCCCGAAGGATTCGCATAACGGTTGCCGGGTCC
CAGGCGCTTGAAGCGGTCTTCTTCCCAATCGTCTCGCCCCGGGTCGGCACGGCGTCAGCGTCCATGCGCTTACAAAGCCCCGTGATGCTGCCCGGGTGAATGGCGGCTTGACTGCCCGGCTTGAAGG
GAAGGTGTTTGTGCGTCTTGATCTCACGCCACCACCACCGGATTACGTCGGGCTCGAACTCGAAGGGTCCGGTAAGGGGAGTGGTCGAGTGCGCAAGCTTGTTGATGACGACATTGACCATTCGGCC
GTTGCGCGTGATCTCCTTCGTCTCCGAAACAAGCTCGAAGCCGTAAGGCGCCTTCCCGCCGACGTACCCGCCCAATTCGCGCTGAAGGTTCTTCGTGTCGAGAATCTTCGCCGACTTCAGCGAAGATT
CTTTGTGCGACGCGTCGAGCCGCATAATCAGGTGAATCAGGTCCATGACGTTTCCCTGCCGGAAGACGCCTTCCTGAGTGGAAACAATCGTCACGCCCAGGGCGAGCAATTCCGAGACAATCGGAAT
CGCGTCCATGACCTTCAGGCGCGAGAAGCGCGACACGTCATAGACAATGATCATGTTGAGCCGCCCGGCGCGGCATTCGTTCAGGATGCGTTCGAACTCCGGGCGCTCCGCCGTCCCGAACGCCGAC
GTGCCCGGCGCTTCGCTGAAATGCCCGACGAACCTGAACCGGCCCCCGTCGCGCTCGACTTCGCGCTGAAGGTCGGCCGCCTTGTCTTCGTTGGCGCTACGCTGTGTCGCTGGGCTTGCTGCGCTCGA
ATTTTCGCGCTCGCGCGACTGACGGTCGTAAGCACCCGCGTACGTGTCCACCCCGGTCACAACCCCTTGTGTCATGTCGGCGACCCTACGCCCCCAACTGAGAGAACTCAAAGGTTACCCCAGTTGGG
GCACTACTCCCGAAAACCGCTTCTGACCTGGGAAAACGTGAAGCCCCGGGGCATCCGCTGAGGGTTGCCGCCGGGGCTTCGGTGTGTCCGTCAGTACGGGCCATAGAGGGGCGTCGTGTAACCCACC
CAGGGCGATTGCTCCGGCACGGGGAAGCCGCGCCACGCCTTCGGGACGTCTGGAATCGCTAGAGCTTGCATGCCTGGAGGTCGACGGATCTTTTCCGCTGCATAACCCTGCTTCGGGGTCATTATAGC
GATTTTTTCGGTATATCCATCCTTTTTCGCACGATATACAGGATTTTGCCAAAGGGTTCGTGTAGACTTTCCTTGGTGTATCCAACGGCGTCAGCCGGGCAGGATAGGTGAAGTAGGCCCACCCGCGA
GCGGGTGTTCCTTCTTCACTGTCCCTTATTCGCACCTGGCGGTGCTCAACGGGAATCCTGCTCTGCGAGGCTGGCCGGCTACCGCCGGCGTAACAGATGAGGGCAAGCGGATGGCTGATGAAACCAA
GCCAACCAGGAAGGGCAGCCCACCTATCAAGGTGTACTGCCTTCCAGACGAACGAAGAGCGATTGAGGAAAAGGCGGCGGCGGCCGGCATGAGCCTGTCGGCCTACCTGCTGGCCGTCGGCCAGGG
CTACAAAATCACGGGCGTCGTGGACTATGAGCACGTCCGCGAGCTGGCCCGCATCAATGGCGACCTGGGCCGCCTGGGCGGCCTGCTGAAACTCTGGCTCACCGACGACCCGCGCACGGCGCGGTTC
GGTGATGCCACGATCCTCGCCCTGCTGGCGAAGATCGAAGAGAAGCAGGACGAGCTTGGCAAGGTCATGATGGGCGTGGTCCGCCCGAGGGCAGAGCCATGACTTTTTTAGCCGCTAAAACGGCCG
GGGGGTGCGCGTGATTGCCAAGCACGTCCCCATGCGCTCCATCAAGAAGAGCGACTTCGCGGAGCTGGTGAAGTACATCACCGACGAGCAAGGCAAGACCGATCCCCGGGGACCTGGAGGTCGACT
CTAGCTAGCTGGCCAGGGCCCGATACGTCGCGGTGAGTTCAGGCTTTTTCATATCTCATTGCCCCCGGACGAGCGTCTGCTCCGCCATTCGCCGTCCGCCGTGCCAATCGGATCAGCCGTCCAAATGC
GGGATTTTCGTTAGTCGGAGGCCAAACGGCATTGAGCGTCAGCATATCATCAGCGAGCTGAAGAAAGACAATCCCCGATCCGCTCCACGTGTTGCCCCAGCAATCAGCGCGACCTTGCCCCTCCAAC
GTCATCTCGTTCTCCGCTCATGAGCTCAGCCAATCGACTGGCGAGCGGCATCGCATTCTTCGCATCCCGCCTCTGGCGGATGCAGGAAGATCAACGGATCTCGGCCCAGTTGACCCAGGGCTGTCGCC
ACAATGTCGCGGGAGCGGATCAACCGAGCAAAGGCATGACCGACTGGACCTTCCTTCTGAAGGCTCTTCTCCTTGAGCCACCTGTCCGCCAAGGCAAAGCGCTCACAGCAGTGGTCATTCTCGAGAT
AATCGACGCGTACCAACTTGCCATCCTGAAGAATGGTGCAGTGTCTCGGCACCCCATAGGGAACCTTTGCCATCAACTCGGCAAGATGCAGCGTCGTGTTGGCATCGTGTCCCACGCCGAGGAGAAG
TACCTGCCCATCGAGTTCATGGACACGGGCGACCGGGCTTGCAGGCGAGTGAGGTGGCAGGGGCAATGGATCAGAGATGATCTGCTCTGCCTGTGGCCCCGCTGCCGCAAAGGCAAATGGATGGGC
GCTGCGCTTTACATTTGGCAGGCGCCAGAATGTGTCAGAGACAACTCCAAGGTCCGGTGTAACGGGCGACGTGGCAGGATCGAACGGCTCGTCGTCCAGACCTGACCACGAGGGCATGACGAGCGT
CCCTCCCGGACCCAGCGCAGCACGCAGGGCCTCGATCAGTCCAAGTGGCCCATCTTCGAGGGGCCGGACGCTACGGAAGGAGCTGTGGACCAGCAGCACACCGCCGGGGGTAACCCCAAGGTTGAG
AAGCTGACCGATGAGCTCGGCTTTTCGCCATTCGTATTGCACGACATTGCACTCCACCGCTGATGACATCAGTCGATCATAGCACGATCAACGGCACTGTTGCAAATAGTCGGTGGTGATAAACTTAT
CATCCCCTTTTGCTGATGGAGCTGCACATGAACCAAAAGGATCTAGGTGAAGATCCTTTTTGATAATCTCATGACCAAAATCCCTTAACGTGAGTTTTCGTTCCACTGAGCGTCAGACCCCGTAGAAA
AGATCAAAGGATCTTCTTGAGATCCTTTTTTTCTGCGCGTAATCTGCTGCTTGCAAACAAAAAAACCACCGCTACCAGCGGTGGTTTGTTTGCCGGATCAAGAGCTACCAACTCTTTTTCCGAAGGTAA
CTGGCTTCAGCAGAGCGCAGATACCAAATACTGTTCTTCTAGTGTAGCCGTAGTTAGGCCACCACTTCAAGAACTCTGTAGCACCGCCTACATACCTCGCTCTGCTAATCCTGTTACCAGTGGCTGCTG
CCAGTGGCGATAAGTCGTGTCTTACCGGGTTGGACTCAAGACGATAGTTACCGGATAAGGCGCAGCGGTCGGGCTGAACGGGGGGTTCGTGCACACAGCCCAGCTTGGAGCGAACGACCTACACCG
AACTGAGATACCTACAGCGTGAGCTATGAGAAAGCGCCACGCTTCCCGAAGGGAGAAAGGCGGACAGGTATCCGGTAAGCGGCAGGGTCGGAACAGGAGAGCGCACGAGGGAGCTTCCAGGGGGA
AACGCCTGGTATCTTTATAGTCCTGTCGGGTTTCGCCACCTCTGACTTGAGCGTCGATTTTTGTGATGCTCGTCAGGGGGGCGGAGCCTATGGAAAAACGCCAGCAACGCGGCCTTTTTACGGTTCCTG
GCCTTTTGCTGGCCTTTTGCTCACATGTTCTTTCCTGCGTTATCCCCTGATTCTGTGGATAACCGTATTACCGCCTTTGAGTGAGCTGATACCGCTCGCCGCAGCCGAACGACCGAGCGCAGCGAGTCA
GTGAGCGAGGAAGCGGAAGAGCGCCCAATACGCAAACCGCCTCTCCCCGCGCGTTGGCCGATTCATTAATGCAGCTGGCACGACAGGTTTCCCGACTGGAAAGCGGGCAGTGAGCGCAACGCAATT
AATGTGAGTTAGCTCACTCATTAGGCACCCCAGGCTTTACACTTTATGCTTCCGGCTCGTATGTTGTGTGGAATTGTGAGCGGATAACAATTTCACACAGGAAACAGCTATGACAAAAAAAAAAAGC
CCCGCGATTGCGGGGCTTTTTTTTTTTGATTACGAATTCGCGGCCGCTTCTAGAGTGTTCACATTCGAACGGTCTCTGCTTTGACAACATGCTGTGCGGTGTTGTAAAGTCGTGGCCAGGAGAATACGA
CAGCGTGCAGGACTGGGGGAGTGCGCATTACTAGGAAGGGCATCTTCGACGAATGAAGGAATCCATCAACCGTCGCGTGGTCATCACCGGAATAGGGATCGTCGCGCCCGATGCCACCGGGGTGAA
ACCGTTCTGGGATCTGCTGACGGCCGGTCGCACCGCGACCCGGACCATCACCGCCTTCGATCCCTCTCCGTTCCGTTCCCGCATCGCCGCGGAATGCGATTTCGACCCGCTTGCCGAAGGGCTGACCC
CCCAGCAGATCCGGCGTATGGACCGGGCCACGCAGTTCGCGGTCGTCAGCGCCCGGGAAAGCCTGGAGGACAGCGGACTCGACCTCGGCGCCCTGGACGCCTCCCGCACCGGCGTGGTCGTCGGCA
GCGCGGTCGGCTGCACCACGAGCCTGGAAGAGGAGTACGCGGTCGTCAGCGACAGCGGCCGGAACTGGCTGGTCGACGACGGCTACGCCGTACCGCACCTATTCGACTACTTCGTGCCCAGCTCCAT
CGCCGCCGAGGTGGCACACGACCGCATCGGCGCGGAGGGCCCCGTCAGCCTCGTGTCGACCGGGTGCACCTCGGGCCTGGACGCCGTGGGCCGCGCGGCCGACCTGATCGCCGAGGGAGCGGCGGA
TGTGATGCTGGCCGGTGCGACCGAGGCGCCCATCTCCCCCATCACCGTGGCGTGCTTCGATGCCATCAAGGCGACCACCCCCCGCAACGACACGCCCGCCGAGGCGTCCCGTCCGTTCGACCGCACC
AGGAACGGGTTCGTACTCGGCGAGGGCGCTGCCGTGTTCGTCCTGGAGGAGTTCGAACACGCGCGCCGCCGGGGCGCGCTCGTGTACGCGGAGATCGCCGGGTTCGCCACTCGCTGCAACGCCTTCC
ACATGACCGGTCTGCGCCCGGACGGGCGGGAGATGGCGGAGGCCATCGGGGTGGCGCTCGCCCAGGCGGGCAAGGCGCCGGCTGACGTCGACTACGTCAACGCCCACGGTTCCGGCACCCGGCAGA

ATGACCGTCACGAGACGGCGGCCTTCAAGCGCAGTCTCGGCGACCACGCCTACCGGGTCCCGGTCAGCAGCATCAAATCCATGATCGGGCACTCGCTGGGCGCGATCGGCTCCCTGGAGATCGCCGC
CTCCGTGCTGGCCATCACACACGACGTGGTGCCGCCCACCGCCAATCTGCACGAGCCGGATCCCGAGTGCGATCTGGACTACGTGCCGCTGCGGGCGCGTGCGTGCCCGGTGGACACGGTGCTCACG
GTGGGCAGCGGGTTCGGCGGTTTCCAGAGCGCCATGGTGCTGTGCGGTCCGGGCTCGCGGGGAAGGTCGGCCGCGTGACGGCCGCCGTGGTGGTGACCGGTCTCGGCGTCGTCGCCCCCACCGGTCT
CGGGGTGCGGGAGCACTGGTCGAGTACGGTCCGGGGGGCGTCGGCGATCGGACCGGTCACCCGGTTCGACGCCGGCCGGTACCCCAGCAAACTGGCCGGAGAGGTGCCCGGTTTCGTCCCGGAGGA
CCATCTGCCCAGCCGGCTGATGCCGCAGACGGACCATATGACGCGCCTGGCGCTCGTCGCGGCGGACTGGGCCTTCCAGGACGCCGCCGTGGACCCGTCGAAGCTGCCGGAGTACGGCGTCGGCGTG
GTCACCGCGAGTTCGGCGGGGGGGTTCGAGTTCGGCCACCGCGAGCTCCAGAACCTGTGGAGCCTGGGCCCGCAGTACGTCAGCGCGTATCAGTCGTTCGCATGGTTCTATGCCGTGAACACCGGTC
AGGTGTCCATCCGGCACGGGCTGCGCGGCCCGGGCGGGGTGCTGGTGACGGAACAGGCGGGCGGCCTGGACGCCCTTGGGCAGGCCCGGCGGCAGTTGCGGCGCGGACTGCCGATGGTGGTCGCGG
GAGCCGTTGACGGCTCGCCCTGCCCCTGGGGCTGGGTGGCGCAGCTCAGCTCGGGCGGCCTCAGCACGTCGGACGACCCGCGCCGGGCCTATCTGCCGTTCGACGCCGCAGCCGGCGGACACGTGCC
GGGAGAGGGCGGCGCCCTGCTCGTCCTGGAGAGCGACGAGTCGGCCCGGGCGCGCGGGGTGACGCGGTGGTACGGGCGCATCGATGGGTACGCCGCCACATTCGACCCCCCGCCCGGTTCGGGGCG
CCCGCCGAACCTGCTGCGGGCCGCGCAGGCGGCACTGGACGACGCGGAGGTCGGACCCGAGGCGGTCGACGTGGTGTTCGCGGACGCGTCCGGCACCCCGGACGAGGACGCGGCGGAGGCCGACG
CGGTGCGGCGCCTGTTCGGACCGTACGGCGTTCCGGTGACGGCGCCGAAGACCATGACCGGCCGCCTCAGCGCGGGCGGCGCGGCCCTCGACGTGGCGACGGCGCTGCTGGCGCTGCGCGAGGGCG
TCGTCCCGCCGACGGTCAACGTCTCCCGGCCGCGGCCGGAGTACGAGCTGGACCTGGTGCTCGCCCCCCGGCGCACGCCCCTGGCCAGGGCCCTGGTGCTCGCGCGGGGCCGGGGCGGGTTCAATGC
GGCGATGGTCGTGGCGGGGCCGCGCGCGGAGACACGGTGAAGCGGCCCGGCGCAGCCGGAGCCGCGGTAAGAGGCCACGGAAGAGAGAGGGATGCGACGGTGAAGCAGCAGCTGACGACGGAAC
GGCTCATGGAGATCATGCGGGAGTGCGCGGGCTACGGTGAGGACGTCGACGCTCTGGGCGACACGGACGGCGCCGACTTCGCCGCACTCGGCTACGACTCGCTGGCGCTCCTGGAAACGGCCGGCC
GGCTCGAGCGCGAGTTCGGCATCCAGCTCGGTGACGAGGTGGTCGCCGACGCCAGGACGCCTGCCGAGCTGACCGCCCTGGTCAACCGGACGGTGGCCGAGGCGGCCTGATACTAGAGTGTTCACA
TTCGAACGGTCTCTGCTTTGACAACATGCTGTGCGGTGTTGTAAAGTCGTGGCCAGGAGAATACGACAGCGTGCAGGACTGGGGGAGTGCGCATTACTAGAAAGAGGAGAAATACTAGATGCCGGA
TACCGACGGCCGGGTGGCGGTCGTGACCGGGGGTACCAGCGGGATCGGCCTGGCAGTAGTCAAGGCGCTCGCCCGACAGGATATGCGTGTCGTCCTGTGCGCCCGCAGCCAGGACGCCGTCGACAG
CACGGTGGAGGAACTGCGGGACGAGAAGCTGGAGGTGGACGGGCGGGCCTGCGACGTGCGTTCGACCGACGACGTCCGCGAGTTCATCGGGTTCGCCACCCGGCGCTTCGGCGGGGTCGACGTCCT
GGTCAACAACGCCGGCCGCAACGGCGGTGGACCCACGGCGCGAATCACGGACGAGCTGTGGCTGGACGTGATCGAGACCAACCTCACCTCGGTCTTCCGCGTCACACGGGAGGTGCTGACGACCGG
TGGCATGCTGGCCGCGGGCAGGGGACGCATCATCAACATCGCCTCCACCGGCGGCAAGCAGGGCGTGGTCCTCGGCGCCCCGTACTCGGCGTCCAAGCACGGCGTCGTCGGCTTCACCAAGGCCCTG
GGCCTCGAACTCGCGAAGTCCGGCATCACCGTGAACGCGGTGTGCCCGGGGTACGTCGAGACGCCGATGGCCGAGCGGGTGCGTCGCGGCTACGCCGAGGTCTGGGACACGTCCGAGGAAGCCGTG
CTGGAGCGGTTCCAGGCCAAGATCCCGCTGGGTCGCTACGCGCAGCCCGAGGAGGTCGCCGGGCTCGTCACCTACCTGACCACCCCCACCGCGGACTCCATCACCGCCCAGGCGCTCAACGTCTGCG
GCGGCCTCGGCAACTACTGATACTAGAGAAAGAGGAGAAATACTAGATGACCGCCGGTGACACGACCCTGACCACCCACCGGACCGAGCACCGGGTGACCGTGAGCGCCCCCGCGCGGCGGGTCTT
CGACCTGGTCGCCGACATCACCGGCTGGCCGCACACCTTCCCCCCCACCGTGCACGCCGAGTACGCGGAACGCGGCGCCTCCGAGGAACGAATCCGCATCTGGGCGACGGCCAACGGGGAGGTCAA
GGCCTGGACCAGCCACCGCTCGCTCGACCGCGAGGGCCTCCGGGTGCGGTTCCGGCAGGAGAAGTCCCAGCACCCCGTCGCGGGCATGGGCGGCGAGTGGATCATCCGGCCGGTCGACGGCGAGCG
GTCGGAGGTCATCCTGACGCACGACTTCCAGGCGGTCGAGGACGACCCGGCCCACGTCGACTGGATCCACCGCGCCGTAGATAGAAACAGCGGTGCGGAGCTGGCCGCCCTGAAGGCCGCGGCGGA
GCGCGCGGACGGCGCGGACGGCACGCTCTTCACGTTCGCGGACGAGGTGACCGTCACCGGAGCACCCGGCGACGTGTACGACTACCTGAACGAGGCCCGTCGCTGGCAGGAACGGCTGCCGCACGT
GGCGGCGGTGTCGCTGGACGAGCCCGCGCCGGGCGTGCAGCGCCTCGGGATGGACACCAAGGGCCCCGACGGCACCGTCCACACGACGGAGTCGGTCCGCATCTGCTTCCCGCAGGACCGGATCGT
CTACAAGCAGTTCACCATGCCGCCGCTGATGGCCGTGCATACCGGGACCTGGCGGATCGCCCCCGCTGCGGACGGGCACGGGACCACGGTCACCTCCCTGCACACCGTCGTCGTGGACGACTCCGCC
GTCACTACGGTGCTCGGCCCGGACGCCACCCTCGCCGACGCCCGGCGCTTCCTGCGCGACGCGCTGGGCCGCAACTCCCGCACCACACTCGGCTTCGCCAAGGAGTACGCCGAGGAGCGCGCGCGCC
GGGCGTGATACTAGAGAAAGAGGAGAAATACTAGATGACCGCCGCCTGGGGCGCCCCGCTGTACCCGCCCTGGATCCCGGCGCGTCCGGGGCGCCGGCGATGCGGCGCGGGGCGGCGGGTCCGCTG
CCCGCCGGTGGAGCCGGCCTCCCGGCCCCGGCAGGAGGGCCGGGTCAGCGTGGTGCCCGCCCTCAGGCAGCCCTCGCCGTCGACCAACCCGGAGGTGCGCGTCCGACTGATCGACCTGTCGTCCCCC
GTGGACTCCTCCCAGTACGAGCCCGACCCGGTGGTGCACGACGTCCTTACGCCGCGCCAGGGCGCGGAACACATGTGCGCCGAGATGCGCGAGCACTTCGGTGTCGAGTTCAGCCCCGACGAACTGC
CGGACGGCGAGTTCCTCTCCCTGGACCGGATCACCCTGACCACCCACACGGGGACGCACGTCGACGCCCCCTCCCACTACGGCAGCCGCGCGCTCTACGGCGACGGGGTGCCGCGGCACATCGACCA
GATGCCGCTCGAGTGGTTCTTCGGCCGCGGCGTGGTGCTGGACCTCACCGACGCGCCCACCGGCACCGTTTCGGCCGCGCGTCTGGAGAAGGAGCTGGCCCGCACCGGCTGTGCGCTGCGCCCCGGC
GACATCGTCCTGCTGCACACCGGTGCCCAGCGCCACGCCGGAACGCCCCGCTACTTCACCGACTTCGCCGGGCTCGACGGCCCAGCCGTACGGATGCTGCTCGACCACGGCGTCCGGGTGATCGGCA
CGGATGCCTTCTCACTCGACGCACCGTTCGGCCACATCATCGACCGGTACCGGGCCACGGGAGACCGCAGCGTGCTGTGGCCCGCCCATGTCGTCGGCCGCGAGCGGGAGTACTGCCAGATCGAGCG
CCTCGCGAACCTGGACCGCCTCCCCGTCTCCTTCGGCTTCCGCGTCTGCTGCTTCCCGGTCAAGGTCGCGGGTGCGGGGGCAGGCTGGACCCGGGCGGTCGCCCTGGTCGACGAGGACTGATACTAG
AGAAAGAGGAGAAATACTAGATGCCGACCCGGGTCAACGACGGCGTCGACGCCGACGAGGTCACCTTCGTCAACCGCTTCACGGTGCACGGGGCGCCCGCGGAGTTCGAGTCGGTCTTCGCCCGGA
CGGCCGCCTTCTTCGCGCGGCAGCCCGGCTTCGTGCGCCACACCCTCCTGCGCGAACGCGACAAGGACAACTCCTACGTGAACATCGCCGTGTGGACCGACCACGACGCGTTCCGGCGTGCGCTCGC
CCAGCCGGGGTTCCTGCCGCACGCCACGGCGCTGCGGGCACTCAGCACGTCCGAGCACGGCCTGTTCACCGCCCGCCAGACCCTGCCCGAGGGCGGCGACACCACCGGCTCGGGTCACCGCTGATAC
TAGAGTGTTCACATTCGAACCGTCTCTGCTTTGACAACATGCTGTGCGGTGTTGTAAAGTCTGGTGTATACTAGAGAAAGAGGAGAAATACTAGATGGACACCTCCACCTACAAGTCCGAGGTGGCC
GCCGCATTCAACTCCGCCGCCGCCACCTACGACCGCCTGGGCGTCGAGTTCTTCACCCCGATGGGCCGCCGGCTGGTCGAGCGGGCCGCCCCGGCCGCCGGTGCCCGCGTCCTGGACGTCGGCTGCG
GCCGCGGTGCCTGCGTCTTCCCGGCCGCCGAGCGGGTCGGCCCCGAGGGCCGCGTCGTCGGCATCGACGTCGCCGAGGCCATGATCGAGGAAGCCACCAAGGAAGCCGCCCTGCGGGACGCCGCCA
TGGTGGAGCTCCGGGTCATGGACGGCGAGCACCCCGACTTCGCCCCGCACTCCTTCGACGTGGTCCTGGGCTCCTACTCCGTCATCTTCCTGCCCGACGCCCCCACCGCCCTGGCCCGCTACGCCCCG
CTGCTGTCCCCGGGTGGCCGCCTGGCCTTCACCTCCCCCGTCTTCTCCGACGACACCTTCCCCTTCCTGCCGCCCGTCTTCACCGAGCTGATCCCCCGGGAGCTGCTGCTGGACCTGCCGCCCCAGTGG
CAGCCCGAGCAGCTCCAGCAGCGCTTCAACTCCTGGCTGGCCGACCCCCGCGACCTGACCCGGACCCTGGAGCGCTGCGGCTTCCAGGAAGTCACCGTGGTGGACGAGCCCGTCACCCTGGTCGCCG
AGTCCGGCCTGGCCTGGGTGGACTGGTCCCACACCCAGGGCATGCGCCTGCTGTGGAACCACCTGGCCCAGGACAAGCGCCAGCAGCTGCGGGAGCGGCTGATCACCTCCCTGGACGCCATGCGGG
ACGGCGACGGCCCGCTGACCATCGACACCCCGGTCCGCTACGTCACCGCCACCGTCCGCAACTGATACTAGAGAAAGAGGAGAAATACTAGATGTCCGAGCAGATCGCCGCCGTCCGTCGCATGGT
GGAGGCCTACAACACCGGCAAGACCGACGACGTCGCCGACTACATCCACCCGGAGTACATGAACCCCGGCACCCTGGAGTTCACCTCCCTGCGGGGCCCCGAGCTGTTCGCCATCAACGTCGCCTGG
GTCAAGAAGACCTTCTCCGAGGAAGCCCGCCTGGAGGAAGTCGGCATCGAGGAGCGGGCCGACTGGGTCCGGGCCCGGCTGGTCCTGTACGGCCGCCACGTGGGCGAGATGGTCGGCATGGCCCCC
ACCGGCCGGCTGTTCTCCGGCGAGCAGATCCACCTGCTGCACTTCGTCGACGGCAAGATCCACCACCACCGGGACTGGCCCGACTACCAGGGCACCTACCGGCAGCTGGGCGAGCCGTGGCCCGAG
ACGGAGCACCGTCGCCCGTGATACTAGAGAAAGAGGAGAAATACTAGATGAACCGCGCCGCCCGTCCGACCGTCCGCGGCATGTCCGCCATCGCCGAGCCCACCGCCCCGCGCGGCGTCATCGTCA
CCGGCGGCGGCACCGGCATCGGCCGCGCCACCGCCCACGCCTTCGCCGACCGCGGCGACCGGGTCCTGGTCGTCGGCCGGACCGCCGCCACCCTGGCCGGCACCGCCGAGGGCCACCCCGGCATCTC
CGTCCTGACCGCCGACCTGACCGACCCCGACGGCCCCCGCGCCATCACCGACGCCGCCCTGGACGCCCTGGGCCGGATCGACGTCCTGGTCAACAACGCCGCCACCGGCGGCTTCGCCGGCCTGGCC
GAGACGGAGCCGGAGGCCGCCCGCGAGCAGTTCGACTCCAACCTGCTGGCCCCGCTGCTGCTGACCCGCCAGACCCTGGACGCCCTGTCCGCCGACGGTGGCGGCACCGTCCTGAACATCGGCTCCG
CCGGCGCCCTGGGCCGTCGCGCCTGGCCGCAGAACGGCGTCTACGGCGCCGCCAAGGCCGGCCTGGACTTCCTGACCCGGACCTGGGCCGTGGAGCTGGCCCCGCGTGGCATCCGGGTCCTGGGCCT
GGCCCCCGGCGTGATCGACACCGGCATCGGCGAGCGCTCCGGCATGTCCCGGGAGGCCTACGCCGGCTTCCTGGGCCAGATCGCCGCCCGGGTCCCGGCCGGTCGGGTCGGTCGCCCGGAGGACATC
GCCTGGTGGGCCGTCCAGCTGGCCGACCCCCGGGCCGCCTACGCCACCGGCGCCGTCCTGGCCGTCGACGGCGGCCTGTCCCTGACCTGATACTAGTAGCGGCCGCTGCAGCCCAAGTCTAAAGTTT
TGTCGTCTTTCCAGACGTTAGTAAATGAATTTTCTGTATGAGGTTTTGCTAAACAACTTTCAACAGTTTCAGCGGAGTGAGAATAGAAAGGAACAACTAAAGGAATTGCGAATAATAATTTTTTCACG
TTGAAAATCTCCAAAAAAAAAGGGCTCCAAAAGGAGCCTTTAATTGTATCGGTTTATCAGCTTGCTTTCGAGGTGAATTTCTTAAACAGCTTGATACCGATAGTTGCGCCGACAATGACAACAACCATC
GCCCACGCATAACCGATATATTCGGTCGCTGAGGCTTGCAGGGAGTCAAAGGCCGCTTTTGCGGGACTTGGCACTGGCCGTCGTTTTACAACGTCGTGACTGGGAAAACCCTGGCGTTACCCAACTTA
ATCGCCTTGCAGCACATCCCCCTTTCGCCAGCTGGCGTAATAGCGAAGAGGCCCGCACCGATCGCCCTTCCCAACAGTTGCGCAGCCTGAATGGCGAATGGCGCCTGATGCGGTATTTTCTCCTTACG
CATCTGTGCGGTATTTCACACCGCATAAATTCCCCAATGTCAAGCACTTCCGGAATCGGGAGCGCGGCCGATGCAAAGTGCCGATAAACATAACGATCTTTGTAGAAACCATCGGCGCAGCTATTTA
CCCGCAGGACATATCCACGCCCTCCTACATCGAAGCTGAAAGCACGAGATTCTTCGCCCTCCGAGAGCTGCATCAGGTCGGAGACGCTGTCGAACTTTTCGATCAGAAACTTCTCGACAGACGTAGA
T

Table S3. ^{1}H NMR (500 MHz) data for **4**, **6**, **7** and **9.**

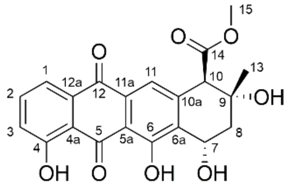
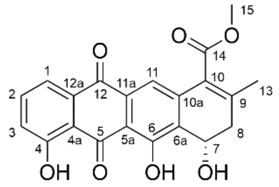
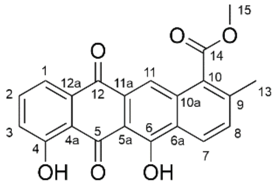
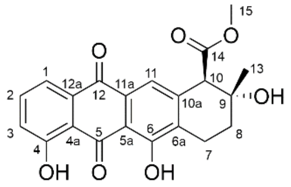

	**6**		**9**		**4**		**7**	
Position	δ^{1}H [ppm]	mult. (J [Hz])	δ^{1}H [ppm]	mult. (J [Hz])	δ^{1}H [ppm]	mult. (J [Hz])	δ^{1}H [ppm]	mult. (J [Hz])
1	7.85	dd (7.5, 1.1)	7.83	dd (7.5, 1.1)	7.90	dd (7.5, 1.1)	7.82	dd (7.5, 1.1)
2	7.71	dd (8.4, 7.5)	7.69	dd (8.4, 7.5)	7.70	dd (8.4, 7.5)	7.67	dd (8.4, 7.5)
3	7.33	dd (8.4, 1.1)	7.31	dd (8.4, 1.1)	7.32	dd (8.4, 1.1)	7.30	dd (8.4, 1.1)
4-OH	11.98	s	12.05	s	12.25	s	12.1	s
6-OH	12.76	s	12.54	s	13.76	s	12.51	s
7	5.39	dd (5.0, 2.0)	5.35	ddd (6.1, 4.9, 2.3)	8.50	dd (8.5, 0.8)	3.05	ddd (-19.1, 6.7, 3.0)
							2.88	ddd (-19.1, 10.3, 6.9)
7-OH			2.23	d (4.9)				
8	2.63	dd (-15.0, 5.0)	2.78	dd (-18.6, 2.3)	7.56	d (8.5)	2.33	ddd (-13.9, 10.3, 6.7)
	2.24	ddd (-15.0, 2.0, 1.4)	2.71	ddd (-18.6, 6.1, 1.2)			1.92	dddd (-13.9, 6.9, 3.0, 1.5)
10	4.06	d (1.4)					3.91	dd (1.5, 0.7)
11	7.72	s	7.67	s	8.26	d (0.8)	7.64	d (0.7)
13-CH_3	1.43	s	2.15	d (1.2)	2.57	s	1.42	s
15-CH_3	3.72	s	3.96	s	4.11	s	3.75	s

Table S4. ^{13}C NMR (125 MHz) data for **4**, **7** and **9**.

	**9**	**4**	**7**
Position	δ^{13}C [ppm]	δ^{13}C [ppm]	δ^{13}C [ppm]
1	120.2	120.1	120.0
2	137.2	137.1	137.1
3	124.7	125.4	124.6
4	162.6	162.7	162.5
4a	115.9	116.7	116.0
5	192.2	191.9	192.9
5a	114.7	108.9	113.6
6	159.9	163.2	161.0
6a	127.7	125.8	133.6
7	59.0	126.2	20.2
8	38.0	131.5	30.8
9	144.3	140.0	69.7
10	126.5	133.0	57.5
10a	133.3	133.4	142.0
11	116.9	118.9	121.2
11a	131.1	129.2	130.8
12	181.4	181.6	181.6
12a	133.6	134.6	133.8
13	22.5	20.6	27.5
14	168.0	168.7	171.7
15	52.3	52.8	52.5

Supplementary figures:

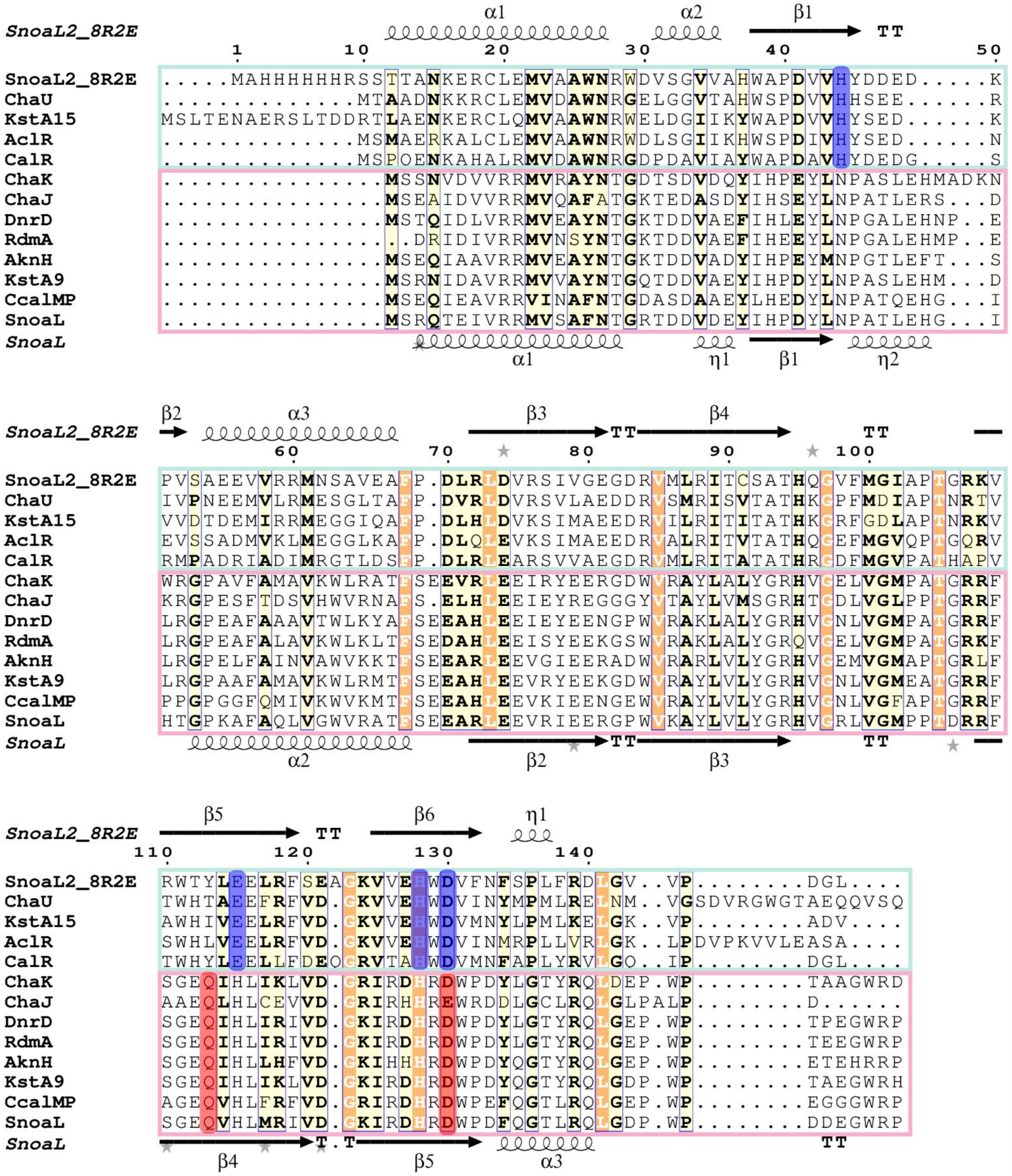

Figure S1. Protein sequence alignment. Sequences are aligned with MUSCLE and ordered based on phylogenetic grouping. Enzymes clustering with SnoaL2 are marked with green border, and the enzymes clustering with SnoaL with pink border. Active site residues are highlighted with blue for SnoaL2 and with red for SnoaL homologs.

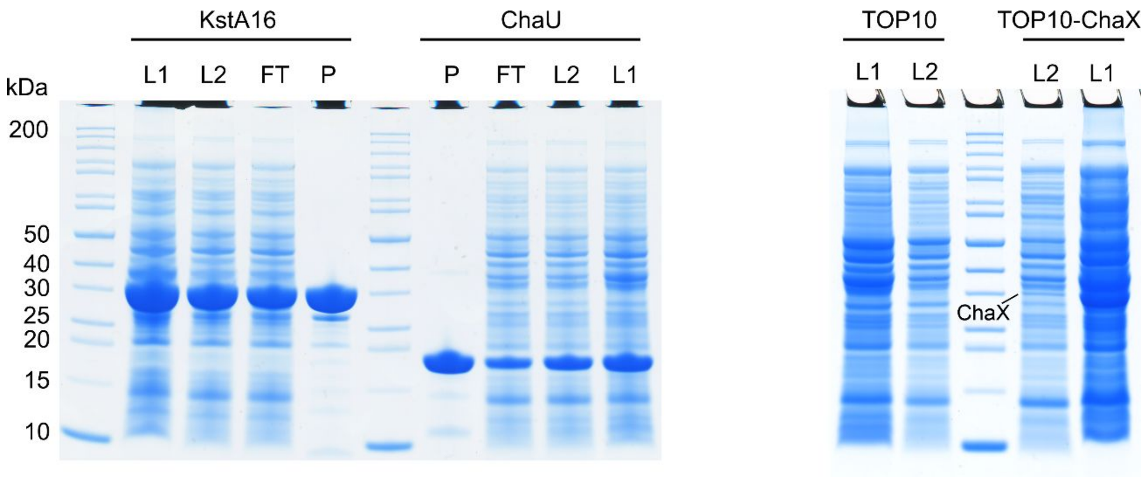

Figure S2. SDS-PAGE. L1: lysate before centrifugation, L2: lysate after centrifugation, FT: flow through, P: purified protein.

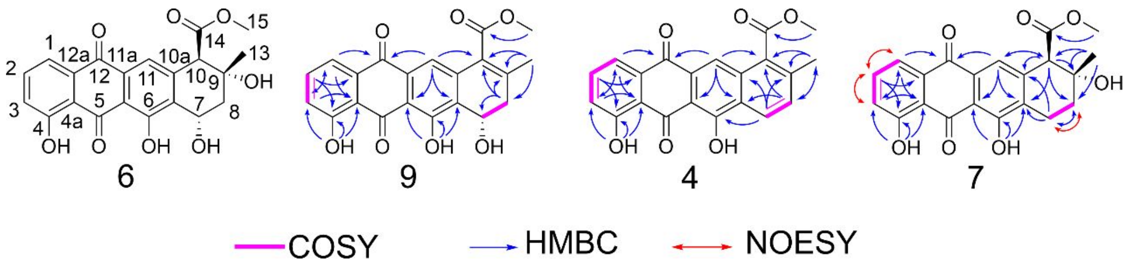

Figure S3. Main HMBC, COSY and NOESY correlations for **6**, **7**, **9** and **4**.

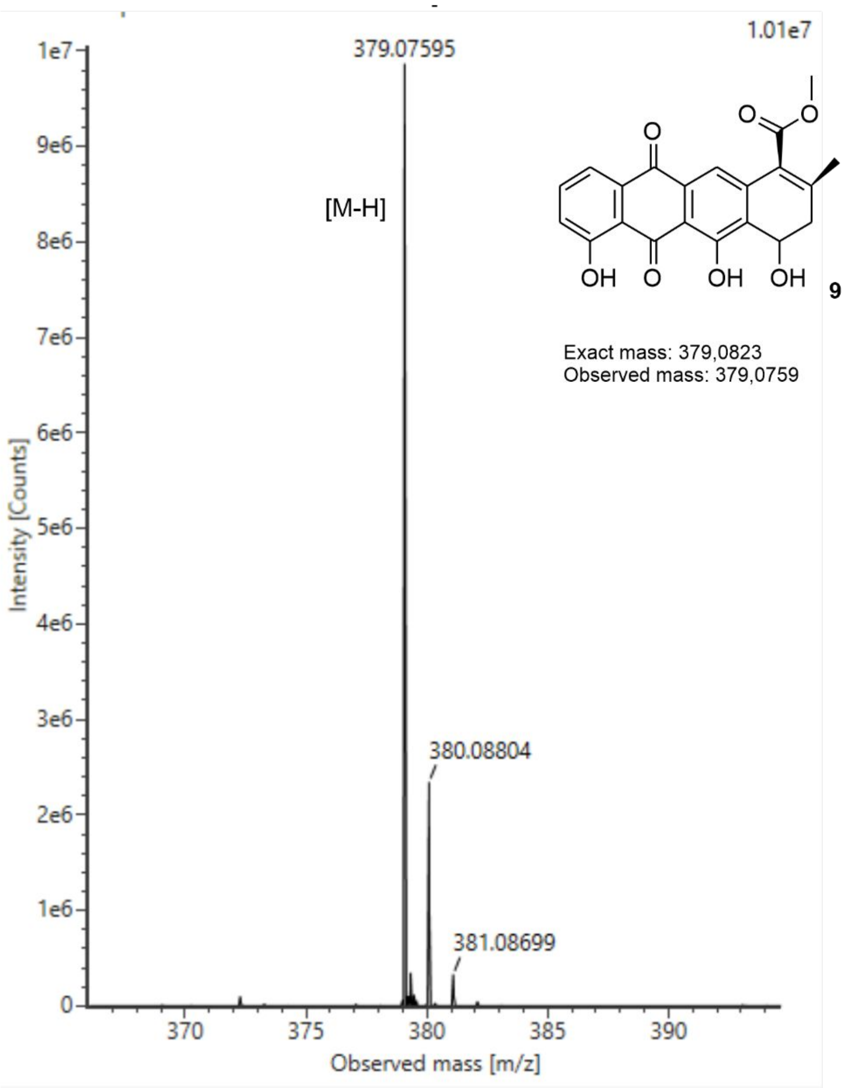

Figure S4. (−)-HRESI-MS of 9. The molecular formula of **9** was verified as C21H18O7 by (−)-HRESI-MS (*m/z* 379.0759 [M − H]⁻ *obs.,* 379.0823 *calc.*

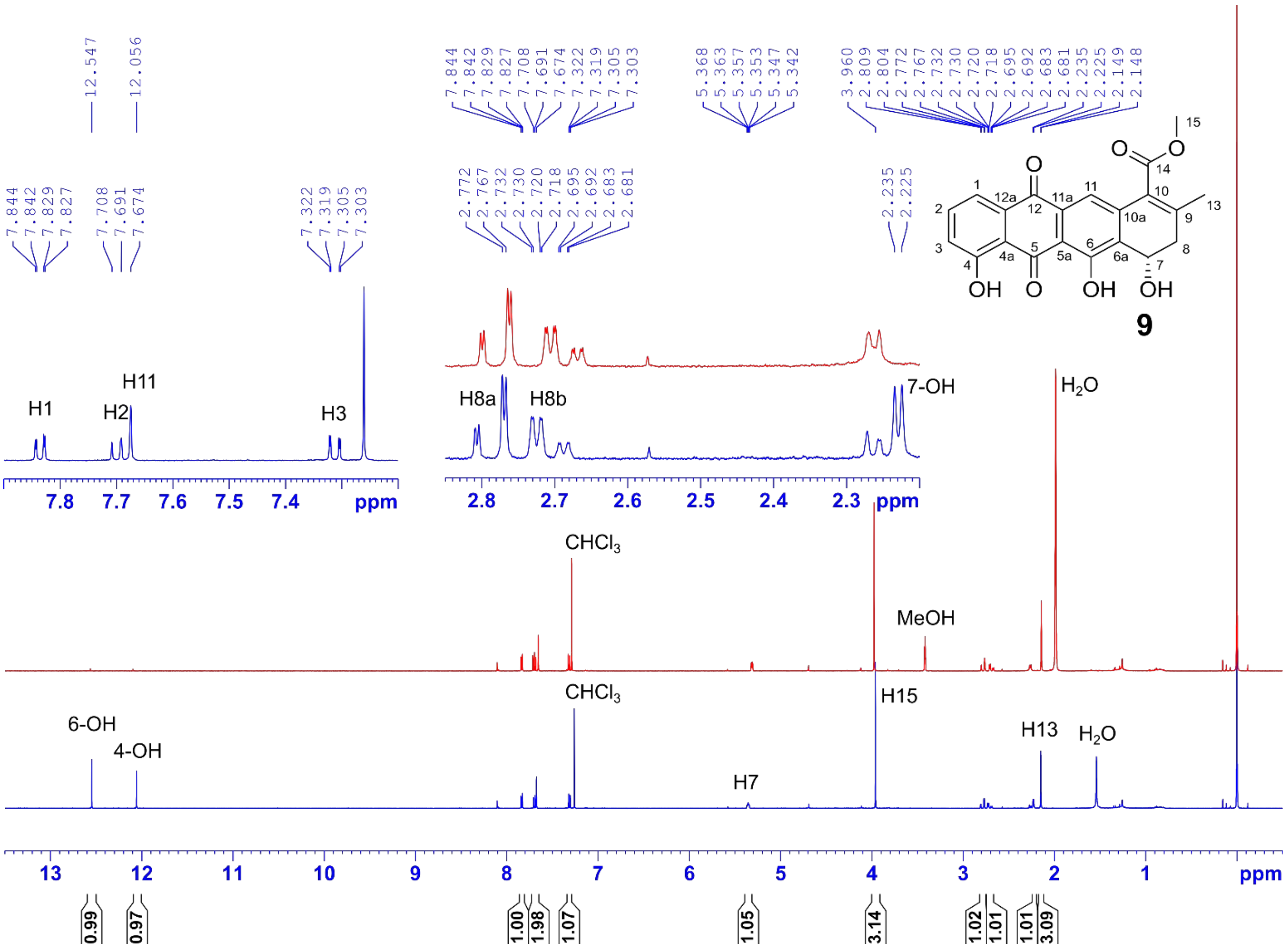

Figure S5. ^{1}H NMR spectrum of **9** in $CDCl_3$, 500 MHz. Blue spectrum in $CDCl_3$, red spectrum in $CDCl_3$+CD_3OD.

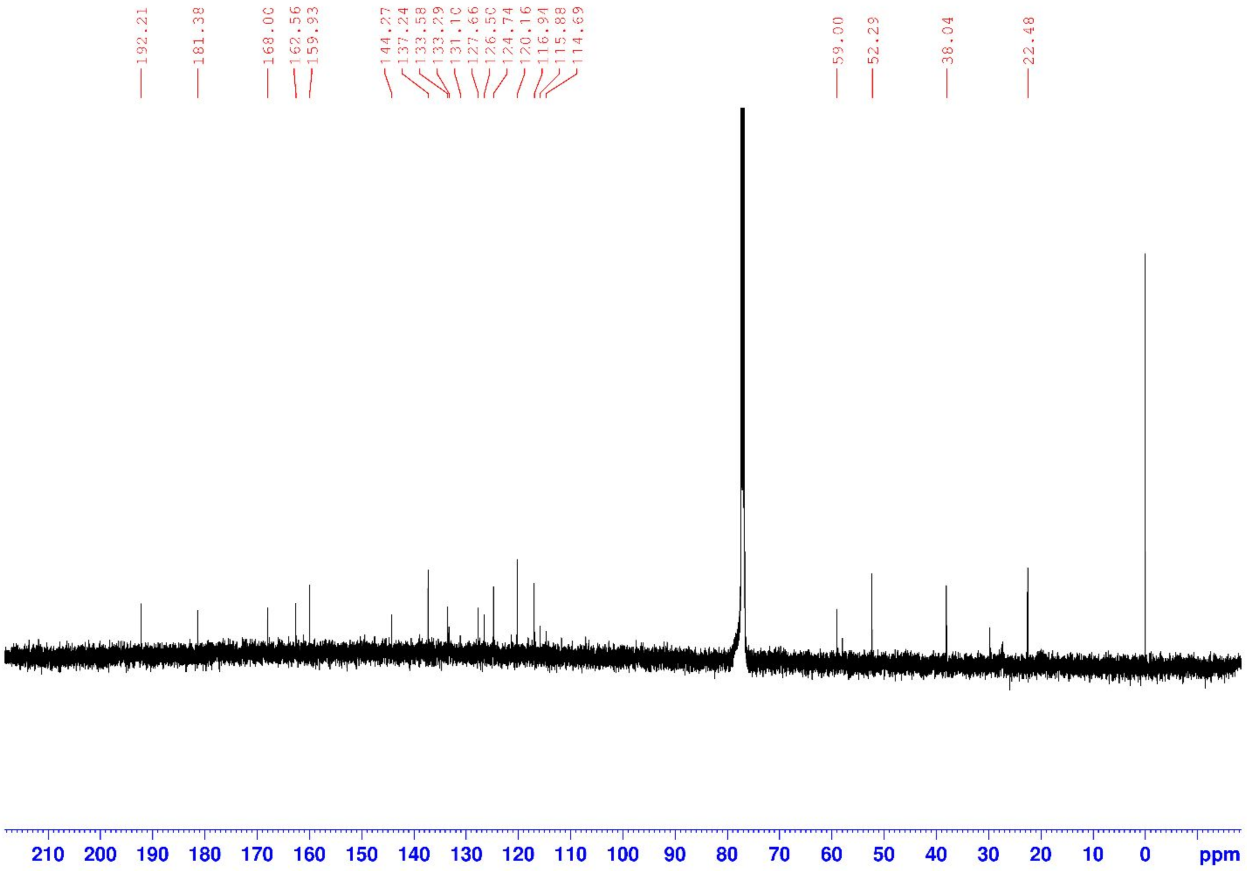

Figure S6. ^{13}C NMR spectrum of **9** in $CDCl_3$

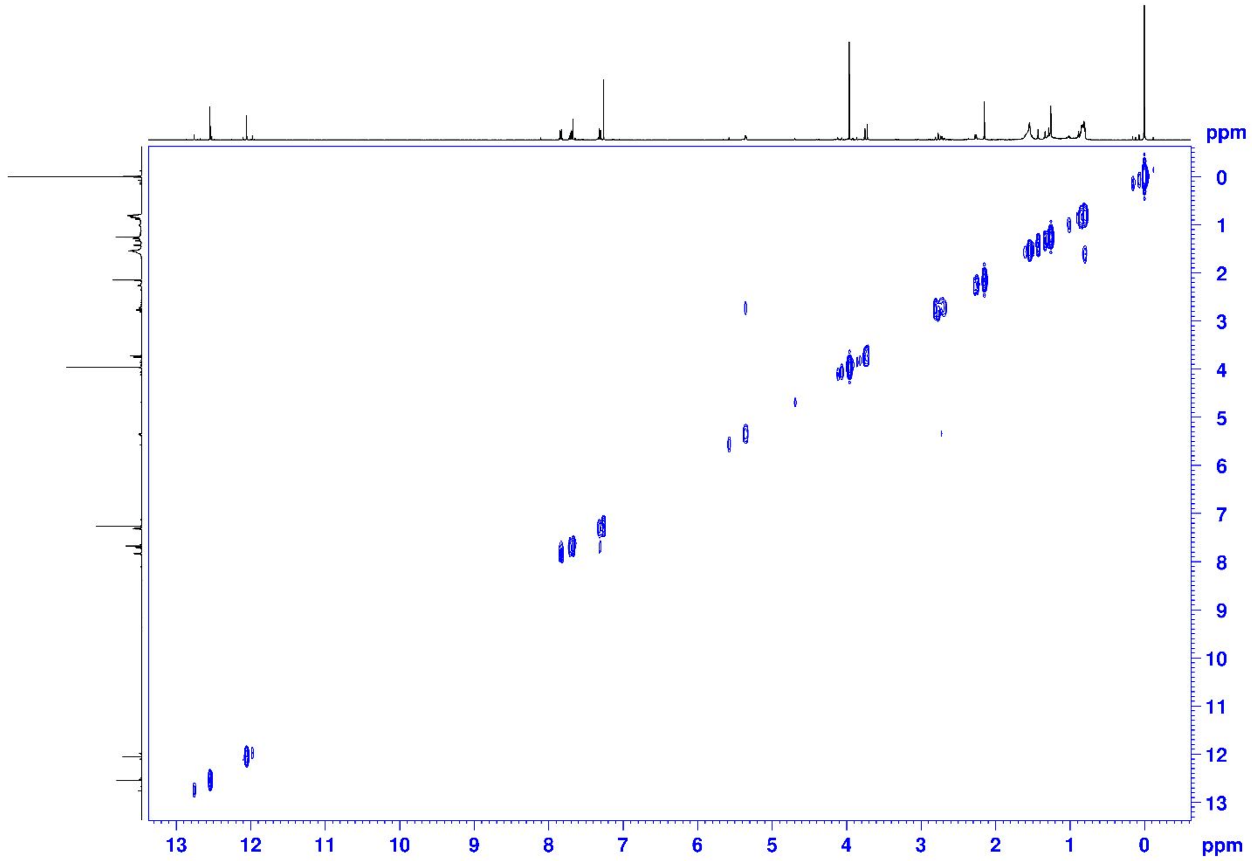

Figure S7. ^{1}H-^{1}H COSY spectrum of **9** in $CDCl_3$

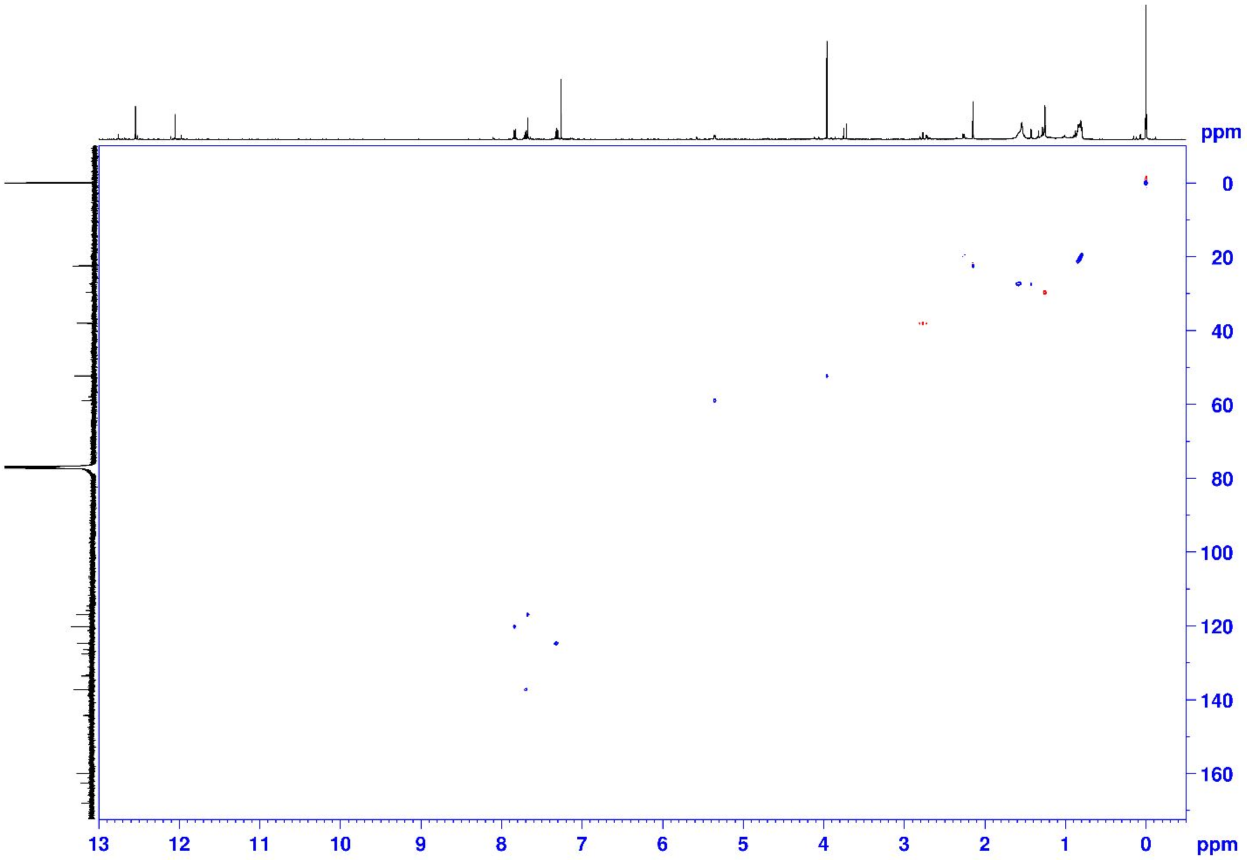

Figure S8. HSQC spectrum of **9** in $CDCl_3$

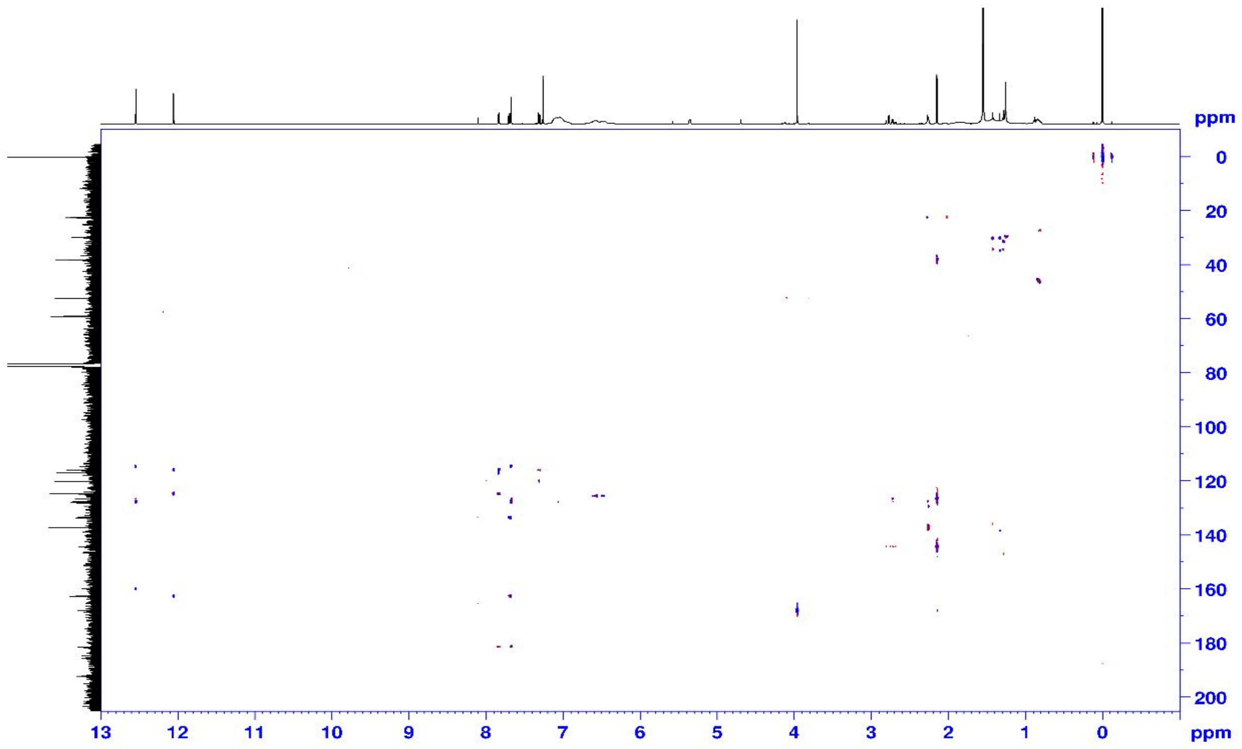

Figure S9. HMBC spectrum of **9** in $CDCl_3$

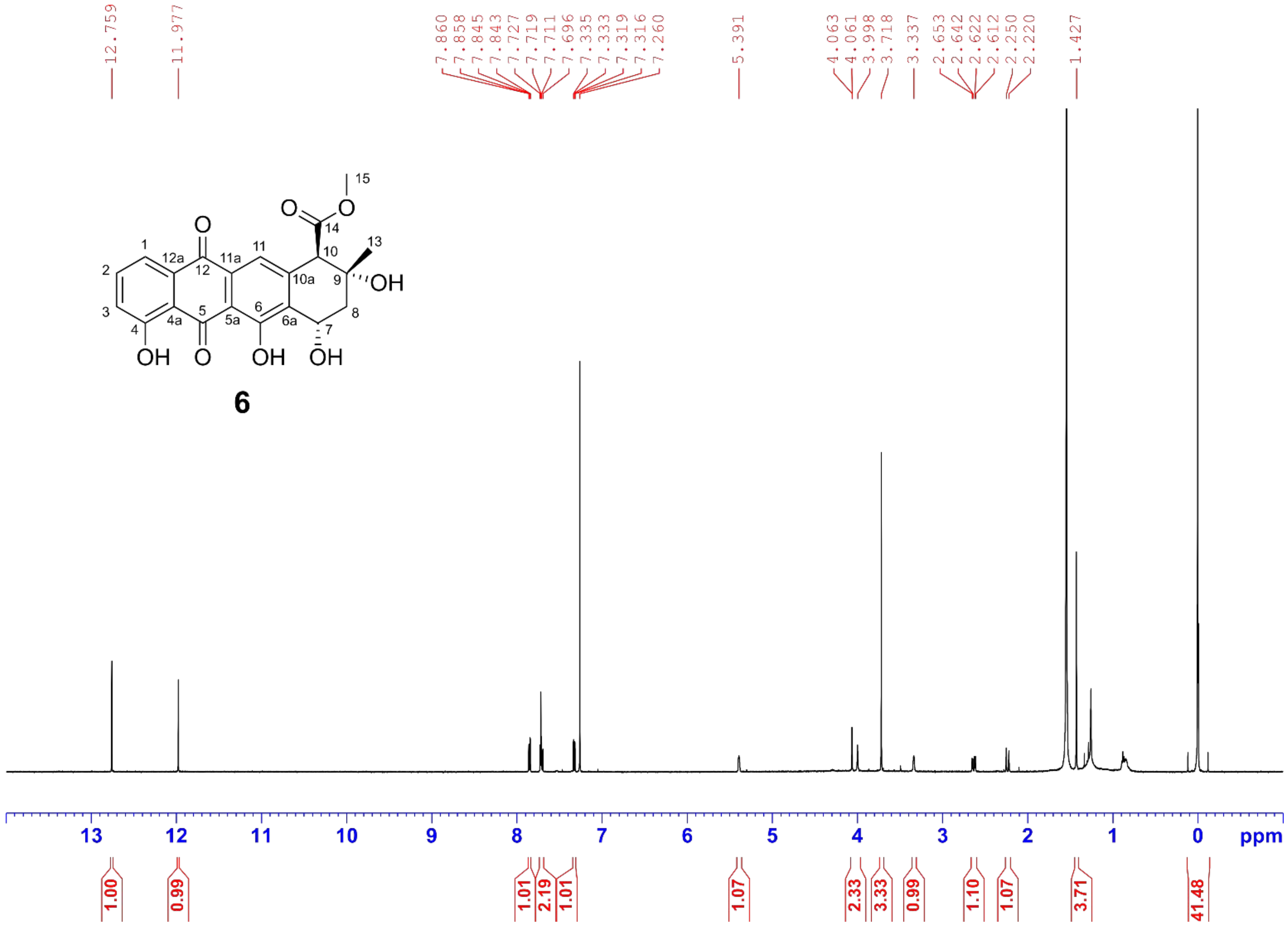

Figure S10. ^{1}H NMR spectrum of **6** in $CDCl_3$, 500 MHz

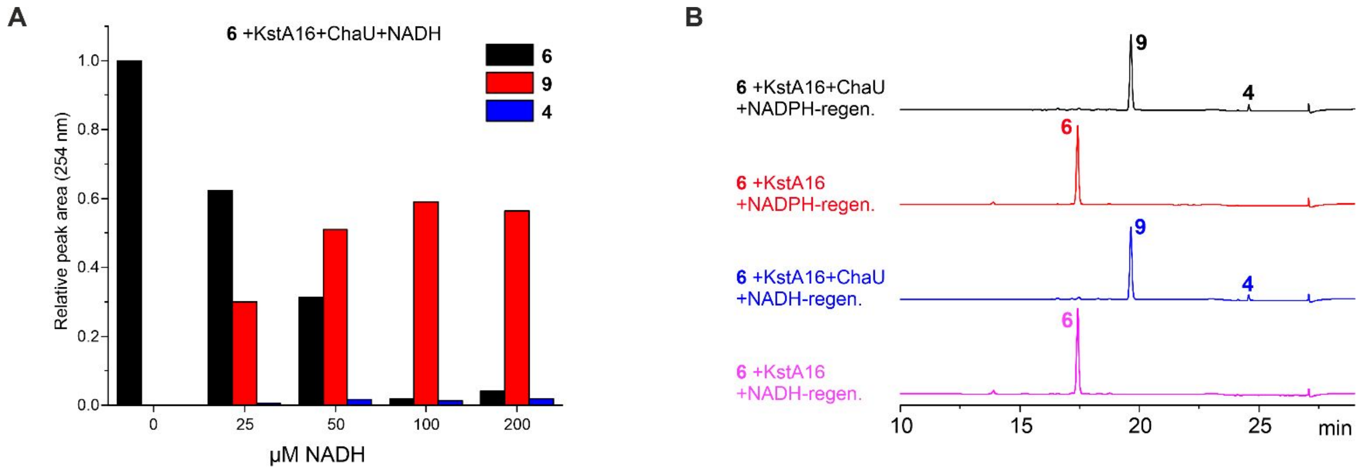

Figure S11. NAD(P)H dependency of KstA16+ChaU reaction. (A) Auramycinone reaction with KstA16 and ChaU in different NADH concentrations. (B) Auramycinone reaction with KstA16 and ChaU with NADH and NADPH with regeneration system (0.5 mM NAD(P)H, 30 mM D-glucose and 10 U/ml glucose dehydrogenase (Sigma-Aldrich)). All chromatograms are measured at 254 nm.

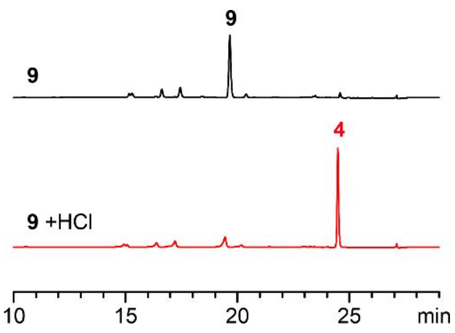

Figure S12. Chemical conversion of 9,10-dehydroauramycinone (9) into resomycin C (4). Enzymatically produced **9** was incubated overnight in 10% MeOH, supplemented with 1% HCl. The end-product (red chromatogram) was extracted with chloroform, dried and analyzed with UPLC. Both chromatograms are recorded at 254 nm.

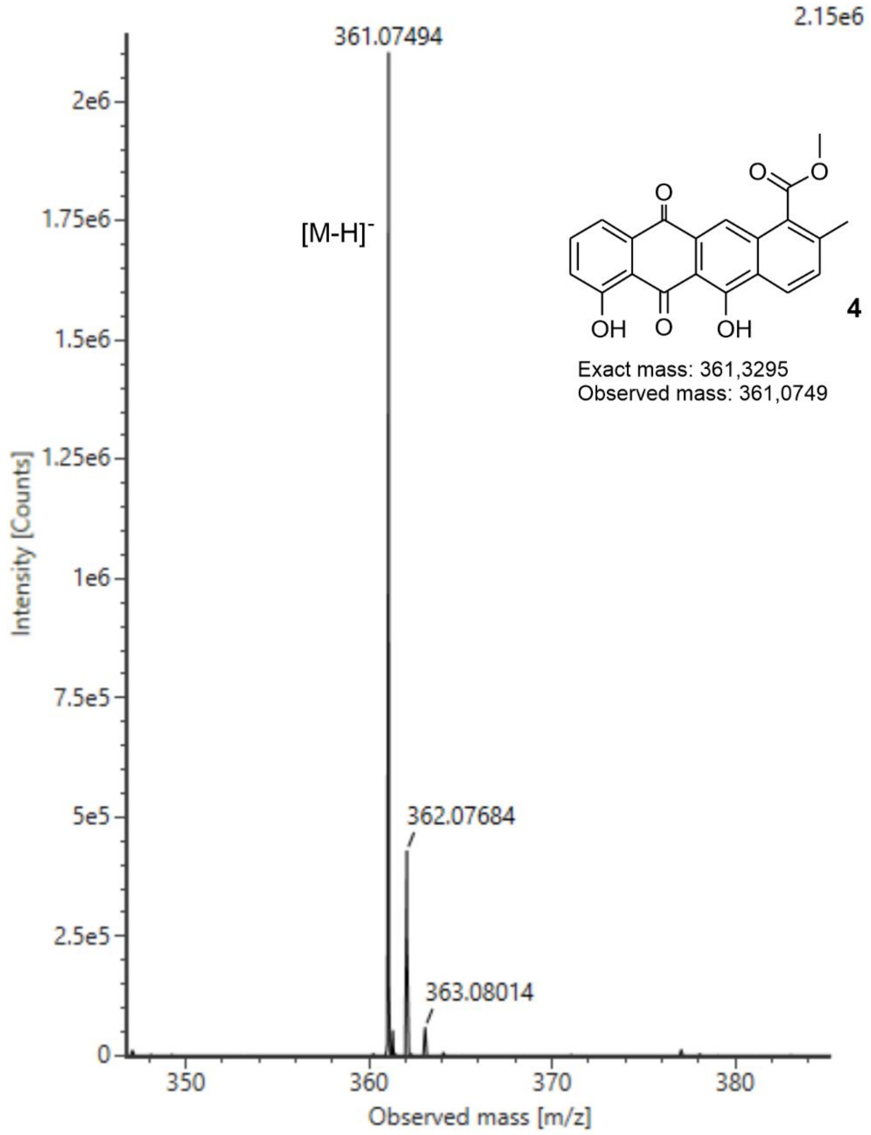

Figure S13. (−)-HRESI-MS of 4. The molecular formula of 4 was verified as $C_{21}H_{18}O_6$ by (−)-HRESI-MS (*m/z* 361.3295 $[M-H]^-$ *obs.,* 361.0749 *calc.*

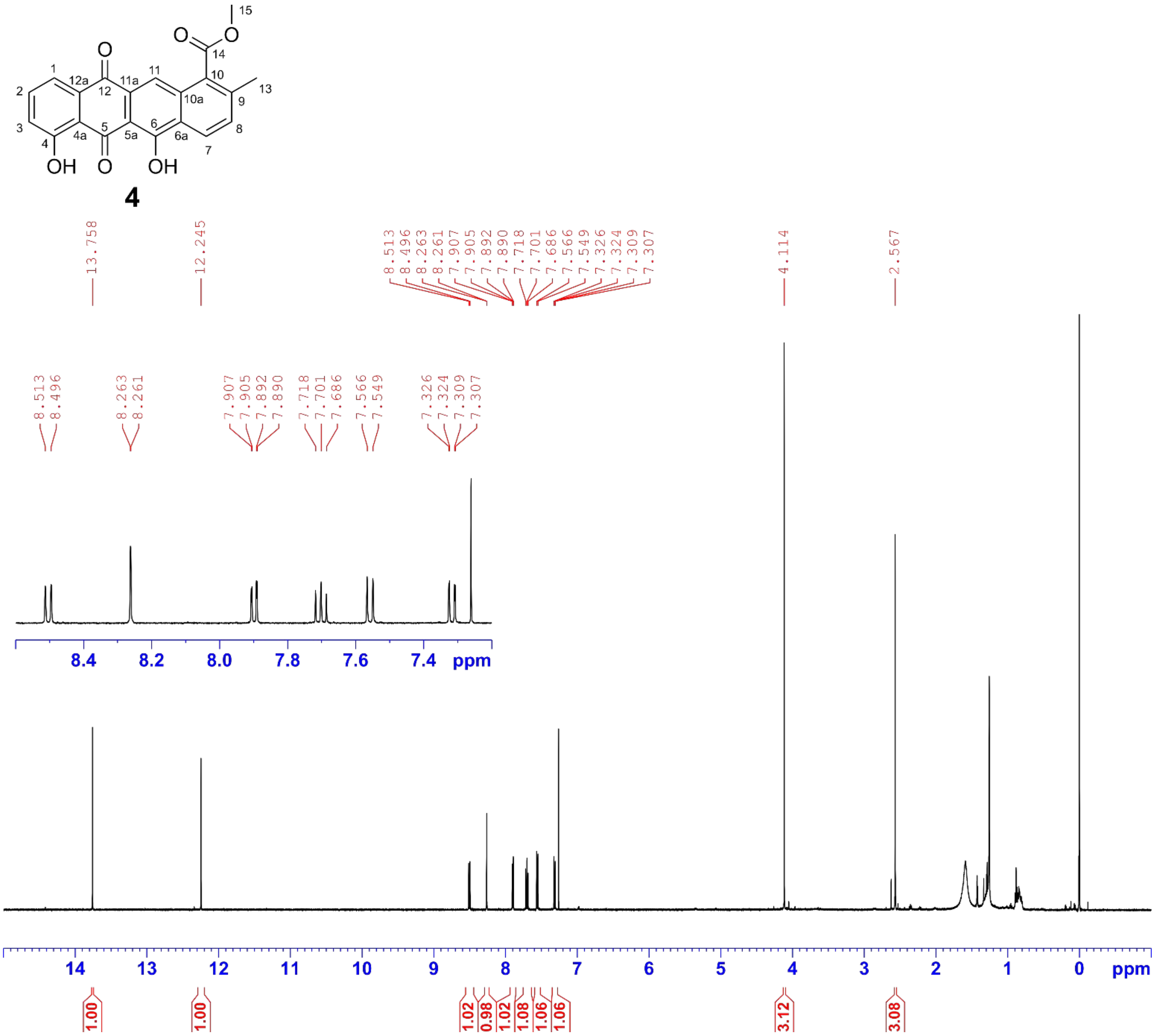

Figure S14. ^{1}H spectrum of **4** in $CDCl_3$, 500 MHz

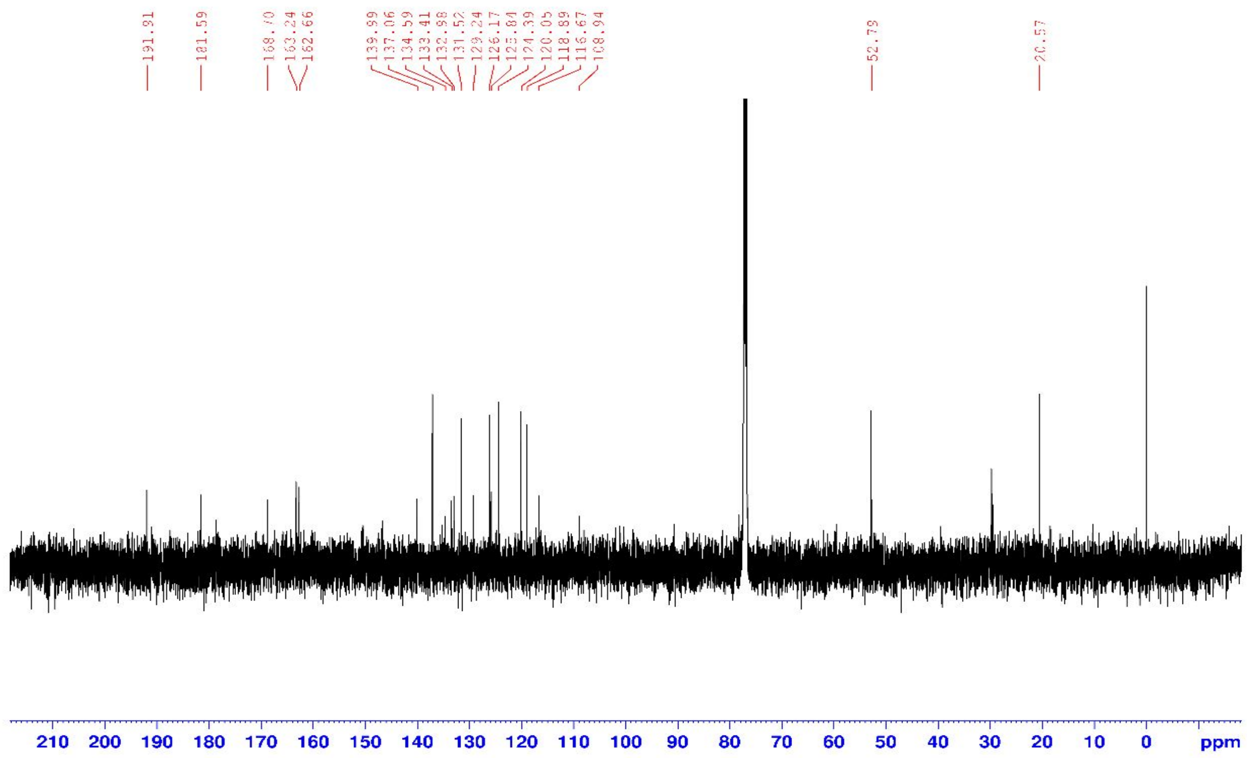

Figure S15. ^{13}C spectrum of **4** in $CDCl_3$

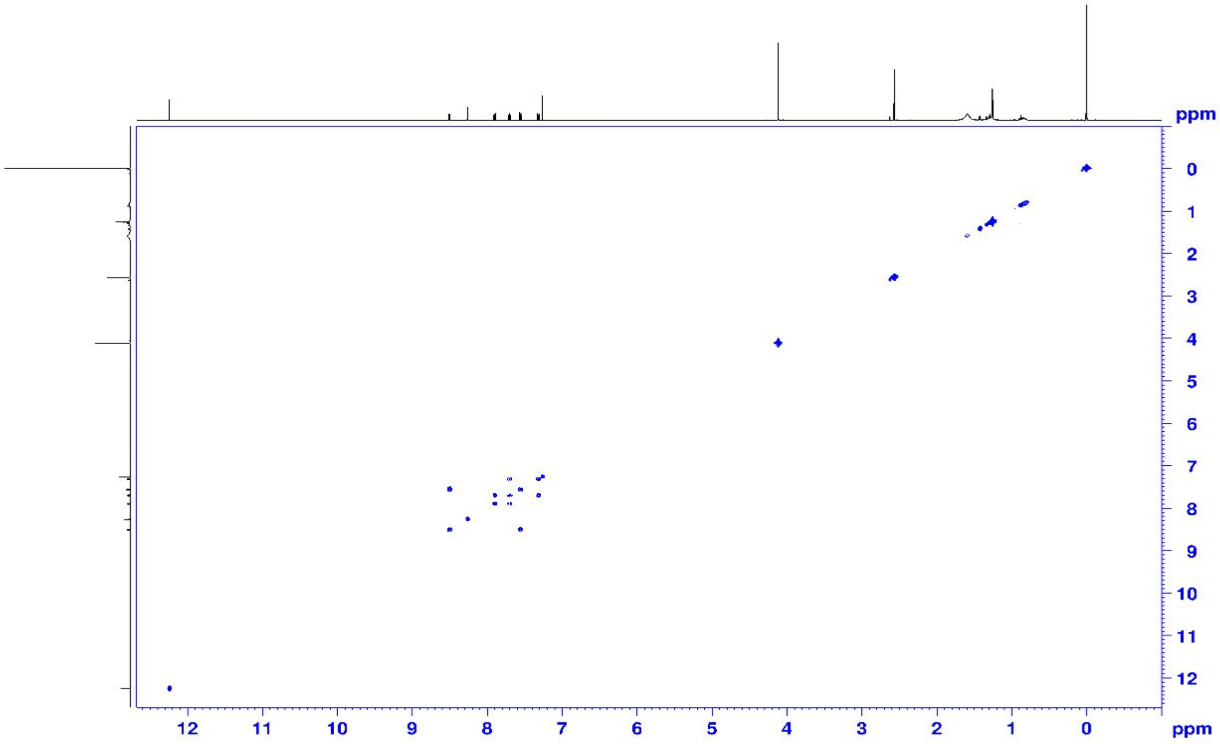

Figure S16. ^{1}H-^{1}H COSY spectrum of **4** in $CDCl_3$

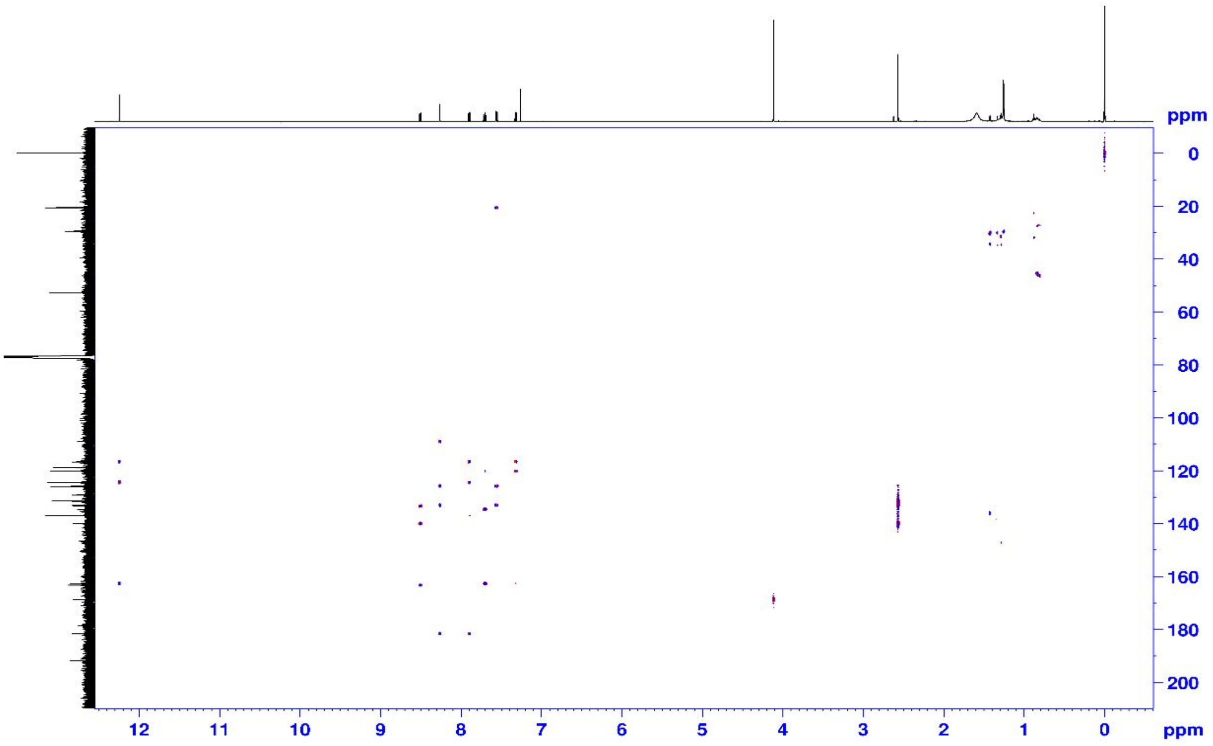

Figure S17. HMBC spectrum of **4** in $CDCl_3$

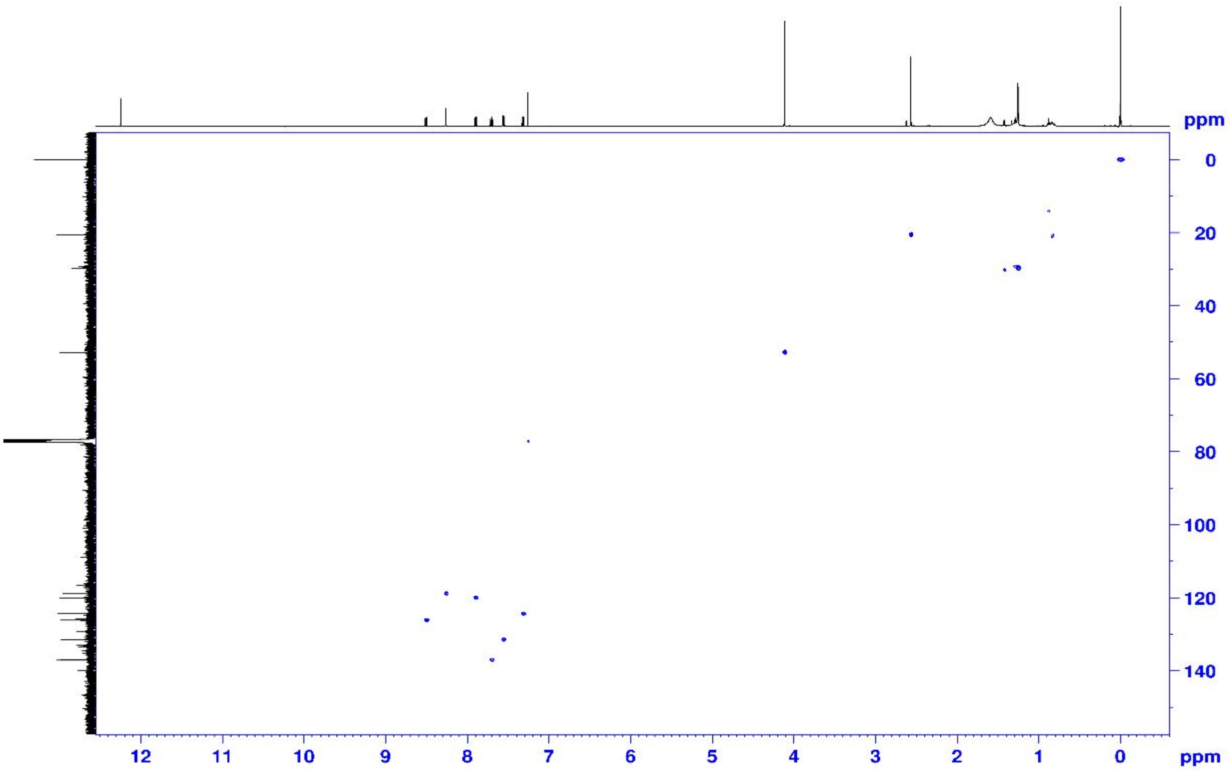

Figure S18. HSQC spectrum of **4** in $CDCl_3$

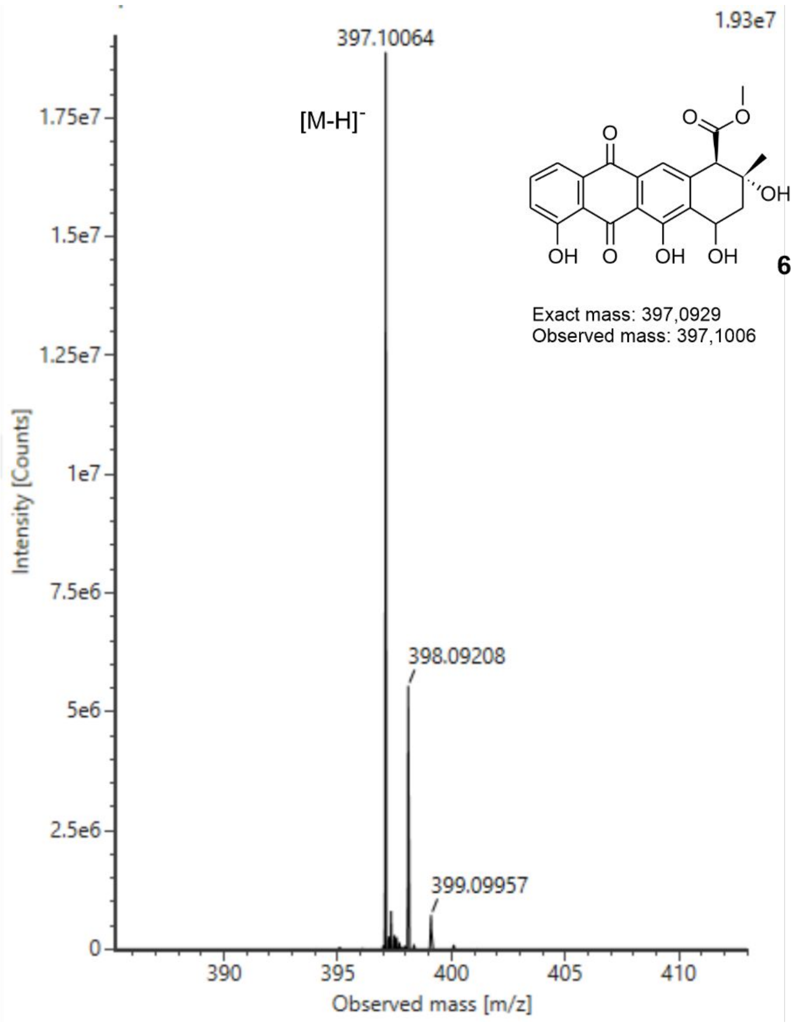

Figure S19. (−)-HRESI-MS of 6. The molecular formula of **6** was verified as $C_{21}H_{18}O_8$ by (−)-HRESI-MS (*m/z* 397.1006 $[M - H]^-$ *obs.,* 397.0929 *calc***.**

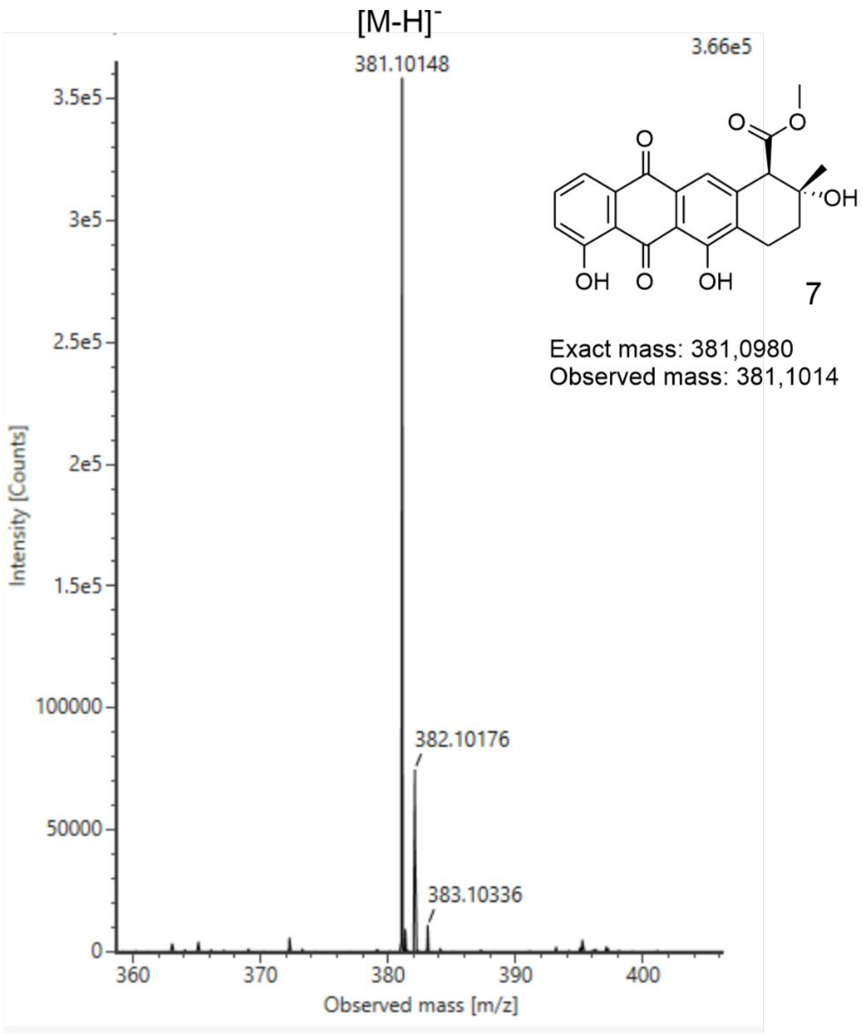

Figure S20. (−)-HRESI-MS of 7. The molecular formula of **7** was verified as $C_{21}H_{18}O_7$ by (−)-HRESI-MS (*m/z* 381.1014 $[M - H]^-$ *obs*., 381.0980 *calc*.

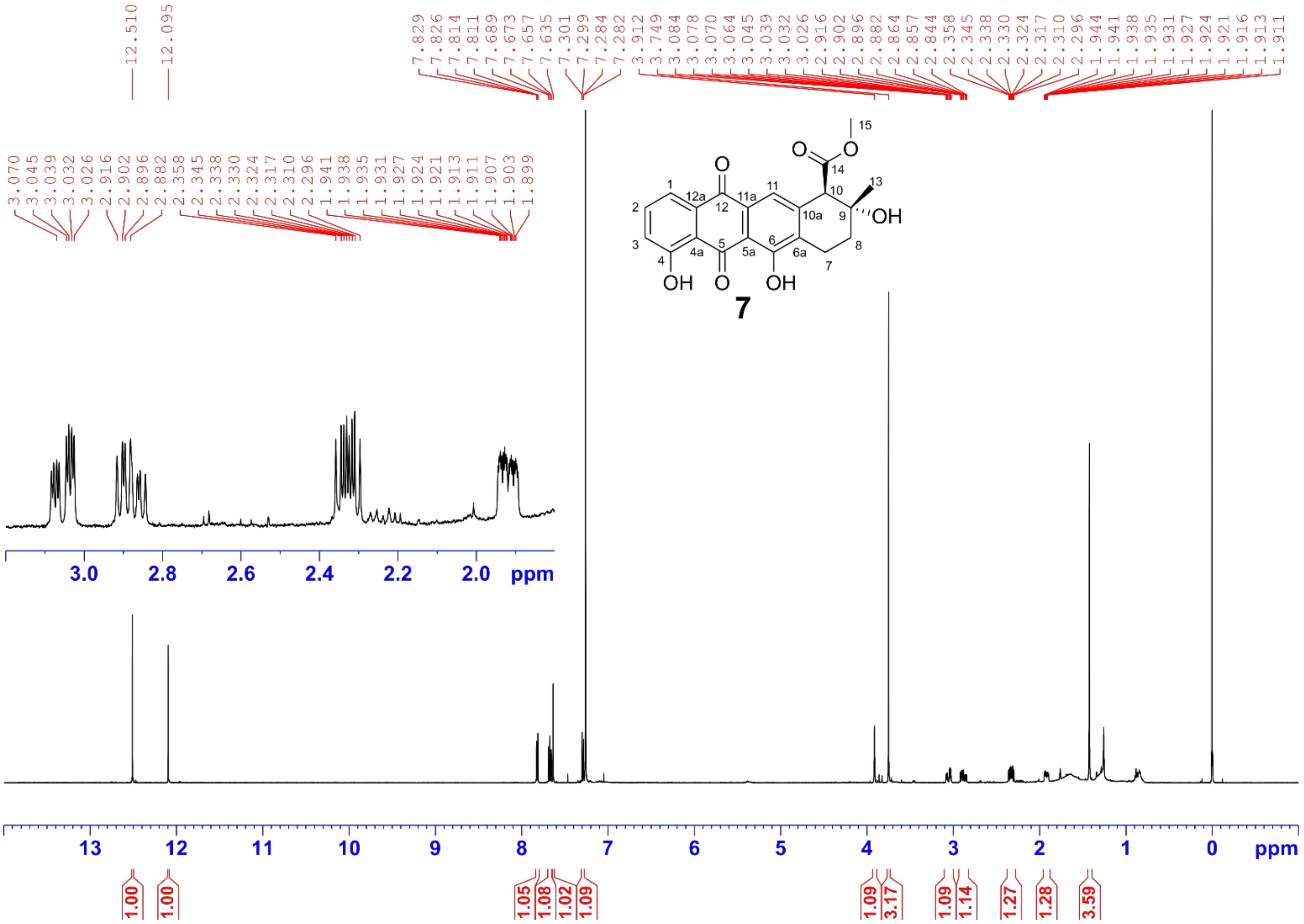

Figure S21. ^{1}H spectrum of **7** in $CDCl_3$, 500 MHz

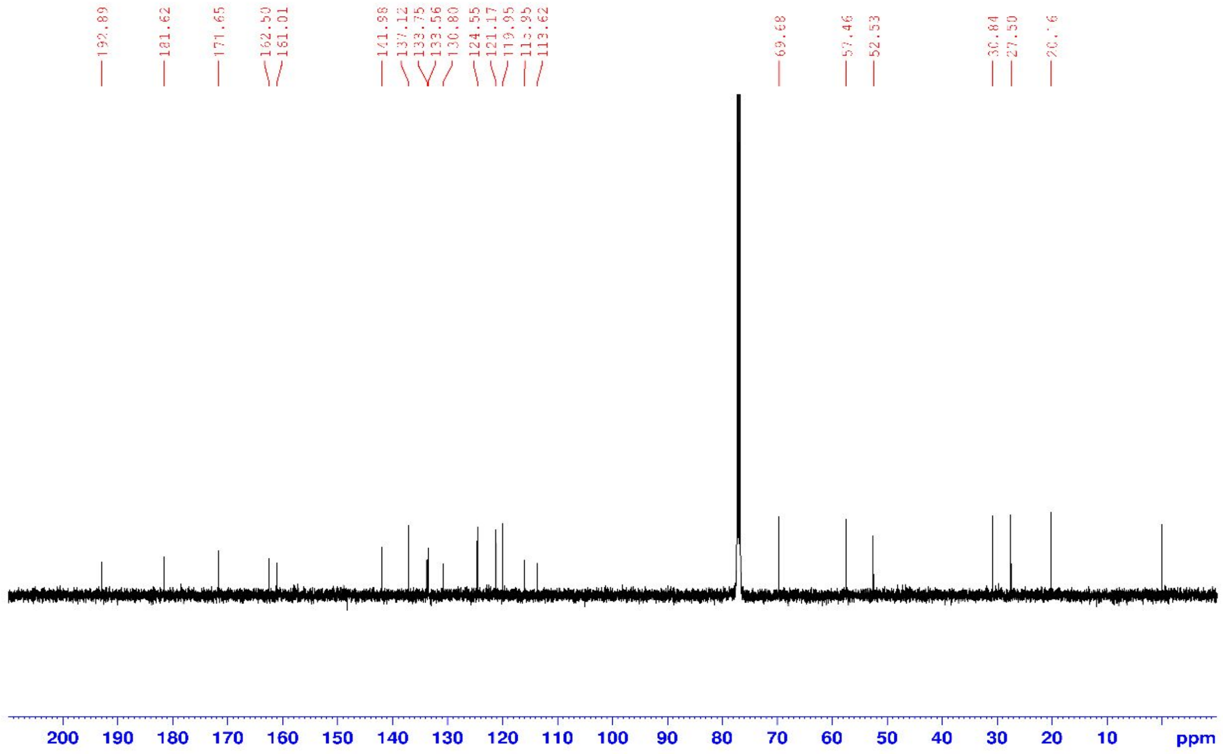

Figure S22. ^{13}C spectrum of **7** in $CDCl_3$

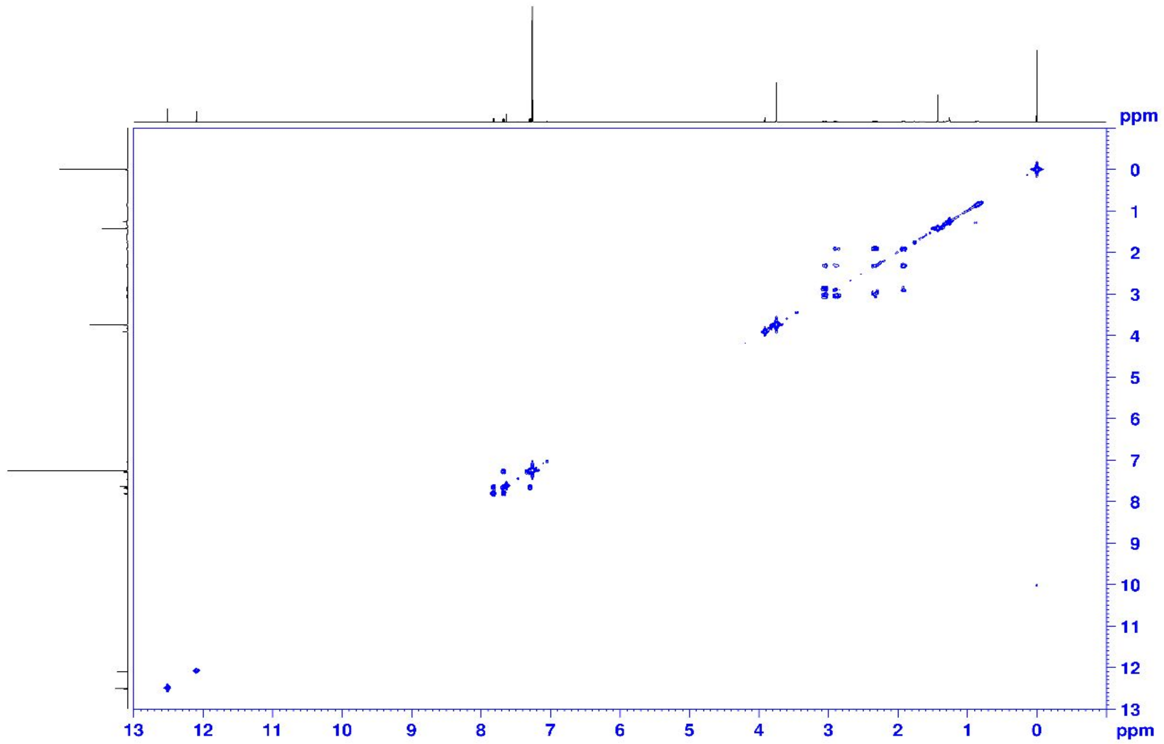

Figure S23. ^{1}H-^{1}H COSY spectrum of **7** in $CDCl_3$

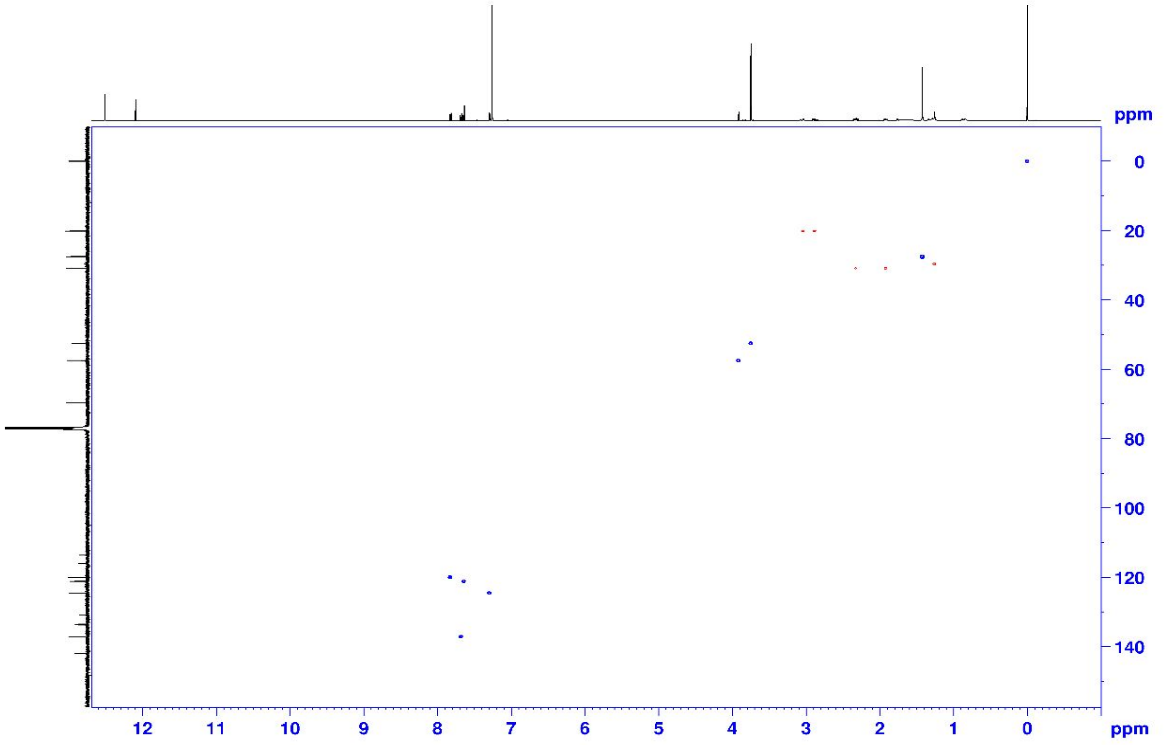

Figure S24. HSQC spectrum of **7** in $CDCl_3$

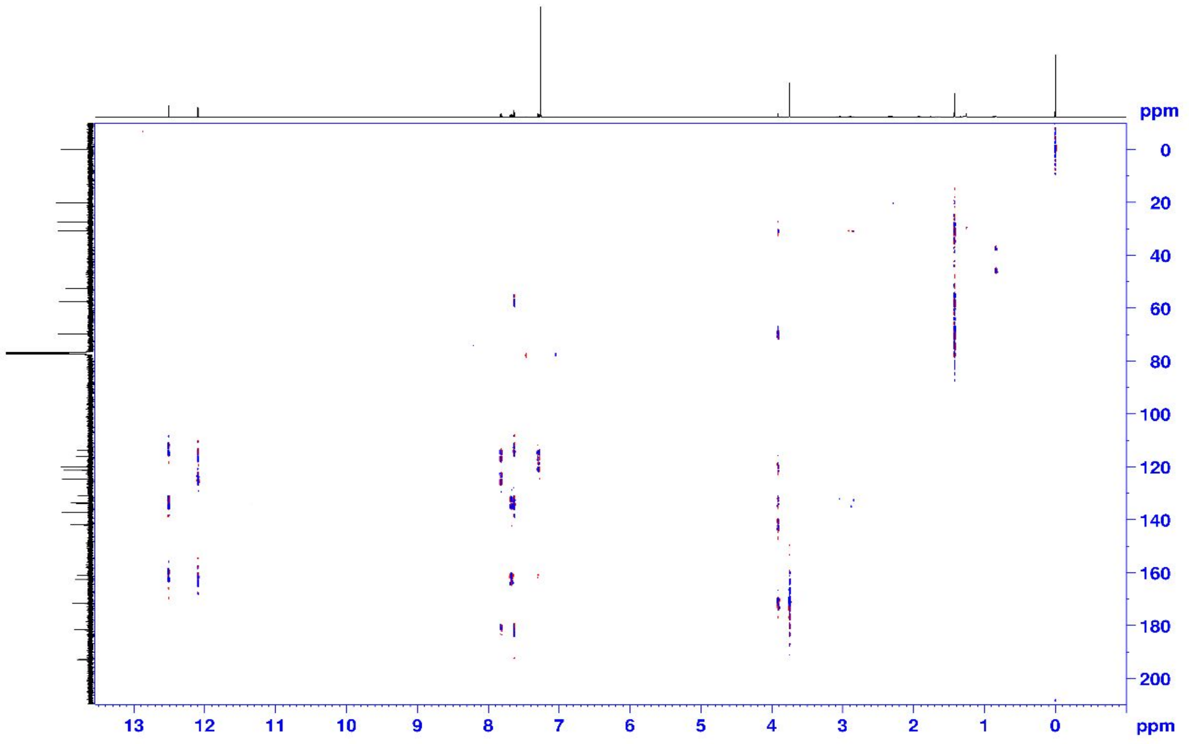

Figure S25. HMBC spectrum of **7** in $CDCl_3$

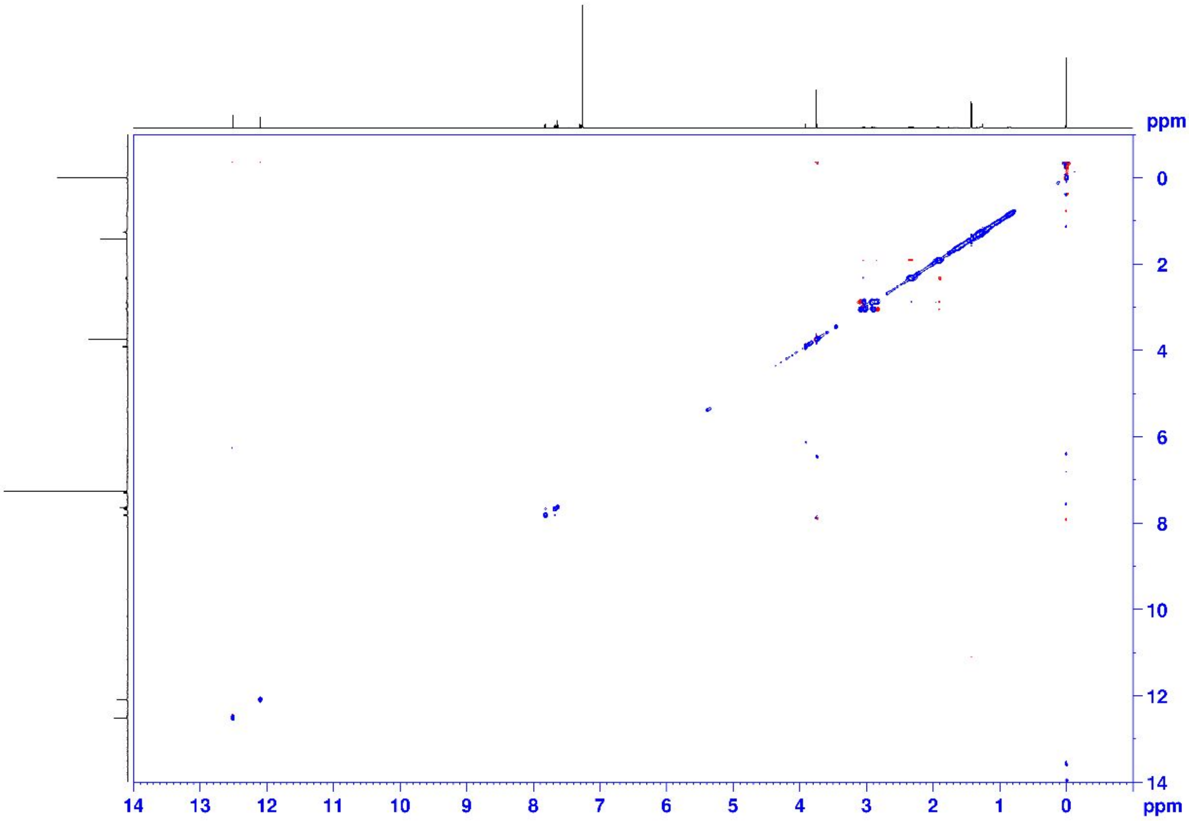

Figure S26. ^{1}H - ^{1}H NOESY spectrum of **7** in $CDCl_3$

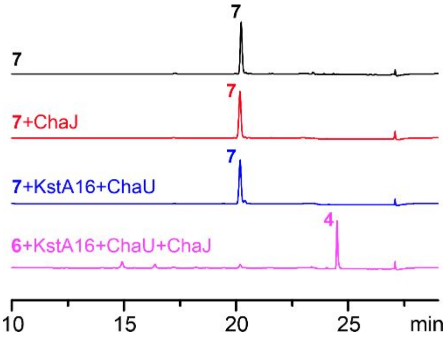

Figure S27. Reactions with the shunt product 7-deoxyauramycinone (7). The reactivity of **7** was tested with KstA16, ChaU and ChaJ in the presence of NADH. As a positive control, **6** was converted into **4** with KstA16, ChaU and ChaJ in the presence of NADH. All chromatograms are recorded at 254 nm.

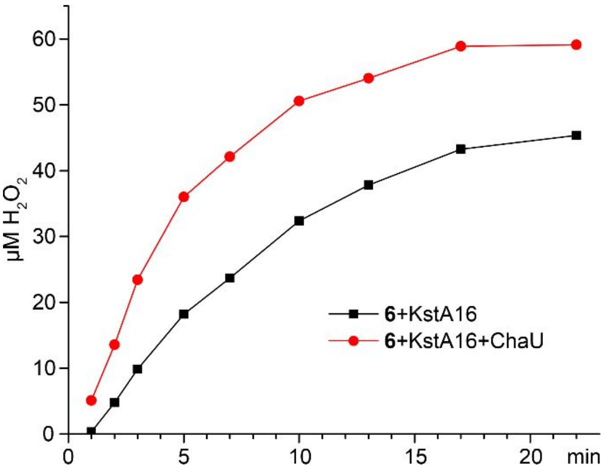

Figure S28. Peroxide formation in auramycinone (6) reactions with KstA16 and ChaU. The formation of peroxides was measured with Pierce Quantitative Peroxide Assay Kit (aqueous-compatible formulation).

References

Wang, R., Nguyen, J., Hecht, J., Schwartz, N., Brown, K. V., Ponomareva, L. V., Niemczura, M., Van Dissel, D., Van Wezel, G. P., Thorson, J. S., Metsä-Ketelä, M., Shaaban, K. A., & Eric Nybo, S. (2022). A BioBricks Metabolic Engineering Platform for the Biosynthesis of Anthracyclinones in Streptomyces coelicolor. *ACS Synthetic Biology*, *11*(12), 4193–4209. https://doi.org/10.1021/acssynbio.2c00498
